# Causal relationship between eosinophilic esophagitis and inflammatory bowel disease: a bidirectional two-sample Mendelian randomization study

**DOI:** 10.3389/fimmu.2024.1374107

**Published:** 2024-04-24

**Authors:** Ruoyu Ji, Yuxiang Zhi

**Affiliations:** Department of Allergy, Peking Union Medical College Hospital, Chinese Academy of Medical Sciences and Peking Union Medical College, Beijing, China

**Keywords:** eosinophilic esophagitis, inflammatory bowel disease, Mendelian randomization, causality, genome-wide association studies

## Abstract

**Background:**

Eosinophilic esophagitis (EoE) and inflammatory bowel diseases (IBDs), including Crohn’s disease (CD) and ulcerative colitis (UC), are immune-mediated gastrointestinal diseases with overlapped pathogenesis and are sometimes concurrently diagnosed, but their causal relationship remains unclear. We investigated the causal relationship between EoE and IBD and its subtypes via a two-sample bidirectional Mendelian randomization (MR) approach.

**Methods:**

MR analyses were performed using summary data of a genome-wide association study (GWAS) on individuals of European ancestry. Independent single-nucleotide polymorphisms correlated with EoE (from a GWAS meta-analysis containing 1,930 cases and 13,634 controls) and IBD (from FinnGen GWASs containing 9,083 IBD, 2,033 CD, and 5,931 UC cases, and GWASs of IBD genetic consortium containing 12,882 IBD, 6,968 UC, and 5,956 CD cases) were selected as instruments. We applied the inverse variance weighted (IVW) method as the primary analysis followed by several sensitivity analyses. For the forward MR study, estimates from IVW methods were subsequently meta-analyzed using a random-effect model.

**Results:**

Our results suggested a causal effect of EoE on IBD [pooled odds ratio (OR), 1.07; 95% confidence interval (CI), 1.02–1.13] and EoE on UC (pooled OR, 1.09, 95% CI, 1.04–1.14). No causal link between EoE and CD was observed (pooled OR, 1.05; 95% CI, 0.96–1.16). The reverse MR analyses revealed no causal effect of IBD (and its subtypes) on EoE. Sensitivity analyses confirmed the robustness of primary results.

**Conclusions:**

Our findings provided evidence of a suggestive causal effect of EoE on IBD (specifically on UC) in the European population. Increased awareness of concurrent or subsequent IBD in patients with EoE is called for. Still, the present evidence is not adequate enough and ought to be validated by further investigations.

## 1 Introduction

Eosinophilic esophagitis (EoE) is a T helper (Th) type 2 cell immune-mediated upper gastrointestinal (GI) disease characterized by esophageal dysfunction clinically and eosinophilic infiltration in the esophageal mucosa pathologically (1). EoE has been recently considered as a late manifestation of the allergic march because of its tight relationship with typical atopic diseases (2). The incidence of EoE is estimated to range from 5 to 10 cases per 100,000 and is still increasing worldwide (3).

Inflammatory bowel disease (IBD), another Th cell-mediated disease of the GI tract with a relatively higher incidence (4), is increasingly concurrently diagnosed with EoE in clinical routines (5). A large-scale prospective cohort study reported that the prevalence of subsequent EoE on primary IBD and subsequent IBD on primary EoE was 980 and 3,322 per 10,0000 persons, respectively (6). The high comorbidity rate is speculated to be due to an overlap in pathogenic mechanisms of the two diseases (7, 8). However, the causal relationship between these two diseases remains largely ambiguous.

Mendelian randomization (MR) is an approach that uses the unique properties of genotype to investigate causal relationships, which offers the advantage of minimizing bias caused by confounding factors and reverse causality (9, 10). With the publication of a well-powered genome-wide association study (GWAS) of EoE in 2022 (11), we performed a bidirectional MR study to investigate the causal relationship between EoE and IBD and its subtypes [ulcerative colitis (UC) and Crohn’s disease (CD)].

## 2 Materials and methods

### 2.1 Data source

**Figure 1:**
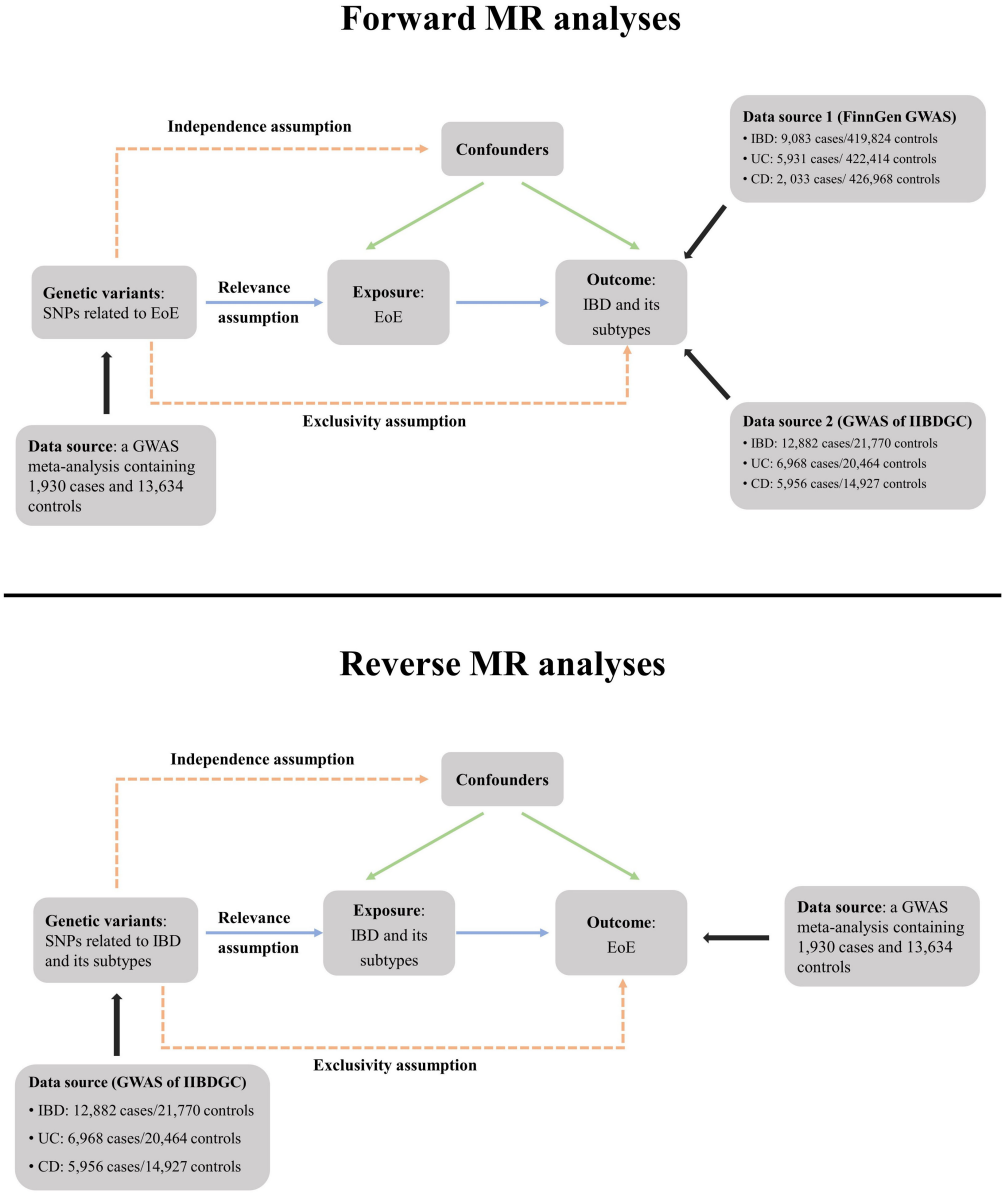
Schematic overview of the study design. In MR analyses, genetic variants must satisfy three principal assumptions to be legitimate instrumental variables (IVs). (1) Relevance assumption, IVs should be strongly associated with the exposure; (2) Exclusivity assumption, IVs should be associated with outcome only through exposure; (3) Independence assumption, IVs should not be associated with confounding factors. MR, Mendelian randomization; SNP, single-nucleotide polymorphisms; EoE, eosinophilic esophagitis; IBD, inflammatory bowel disease; CD, Crohn’s disease; UC, ulcerative colitis; IIBDGC, International IBD Genetics Consortium. GWAS, genome-wide association study.

The EoE dataset originated from a meta-analysis of GWAS (ID GCST90027899) (11). The open-source meta-analysis included 1,930 patients with EoE and 13,634 controls of European ancestry. The study was approved by the corresponding ethics committee and informed consents were collected from all participants. The diagnosis of EoE was both clinically and pathologically confirmed. The GWAS summary data of IBD and its subtypes were obtained from the latest FinnGen datasets (released on 8 December 2023, GWAS IDs: finngen_R10_K11_IBD_STRICT, finngen_R10_K11_CD_STRICT2 and finngen_R10_K11_UC_STRICT2), which contain 9,083 patients with IBD, 2,033 patients with CD, and 5,931 patients with UC of European ancestry, respectively. Detailed information regarding definition of cases, genotype platforms, and statistical analysis protocols is available at the FinnGen website (https:/www.finngen.fi/en/). For validation, we further included three GWAS summary datasets (ieu-a-30, ieu-a-31 and ieu-a-32) published by the International IBD Genetics Consortium (IIBDGC), which contain 5,956 patients with CD, 12,882 patients with IBD, and 6,968 patients with UC of European ancestry, respectively (12) (**Figure 1**). There is no participant overlap between the exposure and outcome datasets.

### 2.2 Selection of the genetic instruments

The forward and reverse MR analyses applied the same criteria for the generation of instrumental variables (IVs). The significant threshold was set as *p* < 5 × 10^−8^ to filter single-nucleotide polymorphisms (SNPs) strongly correlated with the exposure. We further performed a linkage disequilibrium clumping and excluded SNPs with *r* ^2^ ≥ 0.001 and clump distance ≤ 10,000 kb. All selected SNPs were required to have a minor allele frequency (MAF) >1%. We also searched through the PhenoScanner GWAS database (http://phenoscanner.medschl.cam.ac.uk, Version 2) and removed previously reported SNPs (if existed) associated with the outcome and its known confounders under a genome-wide significance threshold of *p* < 5 × 10^−8^ (13). The study flowchart is presented in **Figure 1**. Selected IVs for forward and reverse MR analyses are shown in **Supplementary Tables 1**, **2**, respectively.

### 2.3 MR analyses

MR analyses were performed by using the TwoSampleMR R package and a series of ancillary packages in the R software (Version 4.2.1) (14). We used the random-effect inverse variance weighted (IVW) method to calculate the primary result (15). We used MR-Egger and weighted median methods to test the robustness of our primary result (15, 16). Estimates of individual SNP–exposure correlation versus SNP–outcome correlation were visualized by scatter plots. Heterogeneity was assessed using the Cochran’s *Q* test and was intuitively shown by the leave-one-out analysis (14). When significant heterogeneity was detected, three sensitivity analyses were further conducted. The MR pleiotropy residual sum and outlier (MR-PRESSO) method was utilized to identify and to exclude outliers of instrumental variables with a significant pleiotropic effect (17) (sensitivity analysis A). The MR-Radial analysis was further performed as sensitivity analysis B to identify and to exclude SNPs that were a major source of heterogeneity (18, 19). The sensitivity analysis C excluded SNPs in both sensitivity analysis A and B.

Meta-analyses of MR results were conducted by using a random-effect model in the Revman software (Version 5.3.3). Heterogeneity across MR results was evaluated using the Cochran chi-square and quantified with the *I* ^2^ value. *I* ^2^ values of 25%, 50%, and 75% represent low, moderate, and high heterogeneity, respectively (20). The reverse MR applied similar analytic strategy, and the exposure GWAS of IBD, UC, and CD were ieu-a-31, ieu-a-32, and ieu-a-30, respectively.

## 3 Results

### 3.1 Forward MR analysis

#### 3.1.1 Effect of EoE on IBD

**Figure 2:**
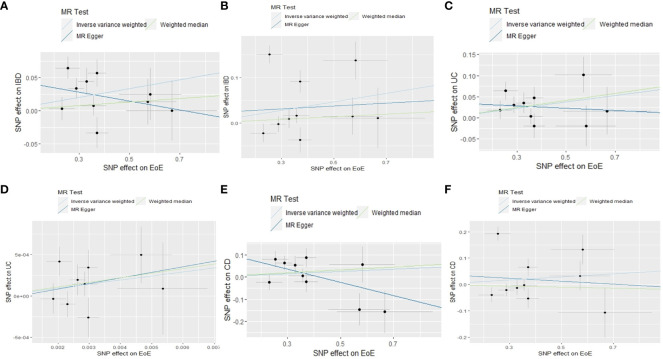
Scatter plots of MR analyses evaluating the causal effect of EoE on IBD based on outcome database of FinnGen **(A)** and IIBDGC **(B)**, EoE on CD based on the outcome database of FinnGen **(C)** and IIBDGC **(D)**, EoE on UC based on the outcome database of FinnGen **(E)** and IIBDGC **(F)**. MR, Mendelian randomization; SNP, single-nucleotide polymorphisms; EoE, eosinophilic esophagitis; IBD, inflammatory bowel disease; CD, Crohn’s disease; UC, ulcerative colitis; IIBDGC, International IBD Genetics Consortium.

Altogether, 15 independent SNPs correlated with EoE were filtered, none of which has been previously identified as the genetic loci associated with IBD, CD,or UC. Ten of the screened SNPs were selected from the FinnGen dataset and five SNPs with a MAF < 1% were excluded (**Supplementary Table 1**). The effect size of each SNP was detailed in **Supplementary Figure 1A**. We used the IVW method to perform the primary analysis. For the MR using the FinnGen dataset, the result indicated a causal relationship between EOE and IBD [odds ratio (OR), 1.07; 95% confidence interval (CI), 1.01–1.13]. We utilized the MR-Egger and weighted median method to test the robustness of our primary result. Results of MR-Egger (OR, 0.95; 95% CI, 0.770–1.14) and weighted median method (OR, 1.03; 95% CI, 0.97–1.08) suggested a non-significant relationship between two diseases. Estimates of individual SNP-EoE correlation versus SNP-IBD correlation were visualized in **Figure 2A**. Significant heterogeneity was detected by Cochran’s *Q* test (*Q* = 17.9; *p* = 0.022) and is visualized by the plot of leave-one-out analysis (**Supplementary Figure 2A**). One pleiotropic outlier was detected by MR-PRESSO (rs56062135, *p* = 0.019). The MR-Radial analysis further identified one outlying SNP. The sensitivity analysis results after removing outliers are demonstrated in **Supplementary Table 3**.

For the MR using the IIBDGC dataset, the same IVs were selected by the IIBDGC dataset (**Supplementary Table 1**). IVW (OR, 1.10; 95% CI, 0.98–1.23), MR-Egger (OR, 1.03; 95% CI, 0.65–1.65), and weighted median (OR, 1.03; 95% CI, 0.97–1.10) methods tested no causal effect of EoE on IBD (**Figure 2B**; **Supplementary Figure 1B**). Heterogeneity was detected (*Q* = 68.2; *p* < 0.001, **Supplementary Figure 2B**). One pleiotropic outlier was detected by MR-PRESSO (*p* < 0.001) and three were detected by MR-Radial. Results of sensitivity analyses were in line with the primary result (**Supplementary Table 3**).

**Figure 3:**
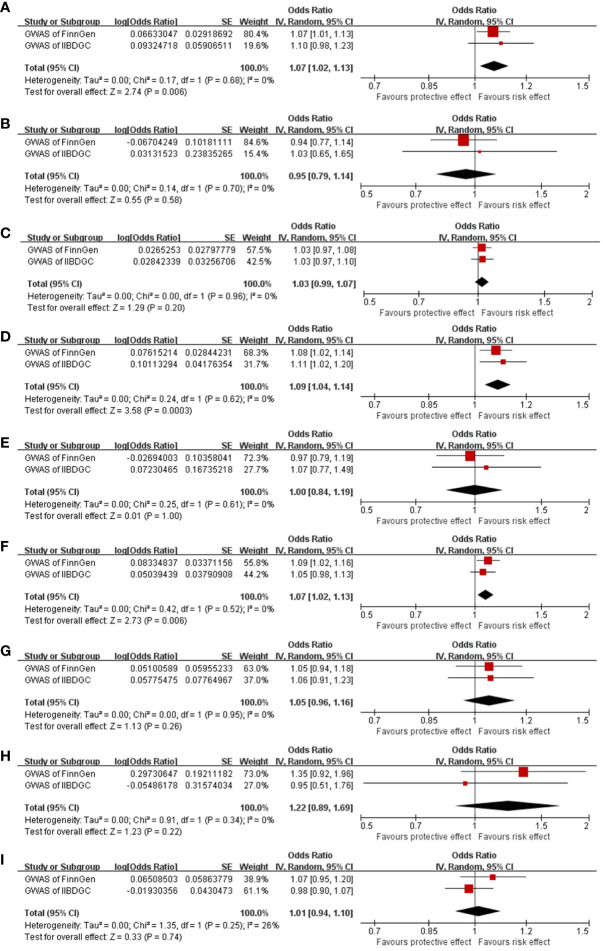
Meta-analysis results of the causal effect of EoE on IBD **(A–C)**, EoE on UC **(D–F)**, and EoE on CD **(G–I)**. Estimated ORs represent the effect of per log-OR increase in EoE on IBD and its subtypes, obtained from results of IVW, MR-Egger, and weighted median methods based on FinnGen and IIBDGC outcome databases separately. EoE, eosinophilic esophagitis; IBD, inflammatory bowel disease; UC, ulcerative colitis; OR, odds ratio; CI, confidence interval; IIBDGC, International IBD Genetics Consortium.

In the meta-analysis of estimates from IVW, the pooled OR was 1.07 (95% CI, 1.02–1.13; *I* ^2 =^ 0, **Figure 3A**). No significant result was obtained from meta-analyses of MR-Egger (OR, 0.95; 95% CI, 0.79–1.14; *I* ^2 =^ 0, **Figure 3B**) and weighted median (OR, 1.03; 95% CI, 0.99–1.07; *I* ^2 =^ 0) methods (**Figure 3C**).

#### 3.1.2 Effect of EoE on UC

According to the selection criteria, 10 of the IVs were available in the FinnGen UC dataset (**Supplementary Table 1**). No pleiotropic outlier was detected by MR-PRESSO (*p* = 0.165). Consistently, results of IVW (OR, 1.08; 95% CI, 1.02–1.14) and weighted median (OR, 1.07; 95% CI, 1.02–1.13) methods indicated a positive causality of EoE on UC (**Figure 2C**; **Supplementary Figure 1C**). The result of MR-Egger is not significant (OR, 0.97; 95% CI, 0.79–1.19). No statistical heterogeneity was indicated (*Q* = 12.2; *p* = 0.143, **Supplementary Figure 2C**).

Ten of the screened IVs were available in the IIBDGC dataset (**Supplementary Table 1**). The IVW suggested a causal effect of EoE on UC (OR, 1.11; 95% CI, 1.02–1.20). Results of MR-Egger (OR, 1.07; 95% CI, 0.77–1.49) and weighted median (OR, 1.05; 95% CI, 0.98–1.13) methods were not significant (**Figure 2D**; **Supplementary Figure 1D**). Heterogeneity was detected (*Q* = 21.6; *p* = 0.001, **Supplementary Figure 2D**). The rs56062135 was identified as an outlier concurrently by MR-PRESSO (*p* = 0.016) and MR-Radial. The sensitivity analysis did not alter the result (**Supplementary Table 3**).

In the meta-analysis of estimates from IVW, the pooled OR was 1.09 (95% CI, 1.04–1.14; *I* ^2 =^ 0%). Meta-analyses of weighted median generated a similar result (OR 1.07; 95% CI, 1.02–1.13; *I* ^2 =^ 0). Meta-analysis of MR-Egger suggested an absence of causal effect (OR, 1.00; 95% CI, 0.84–1.19; *I* ^2 =^ 0) (**Figures 3D–F**).

#### 3.1.3 Effect of EoE on CD

Ten IVs in the FinnGen CD dataset met the selection criteria (**Supplementary Table 1**). Consistently, results of IVW (OR, 1.05; 95% CI, 0.94–1.18), MR-Egger (OR, 1.35; 95% CI, 0.92–1.96), and weighted median (OR, 1.07; 95% CI, 0.95–1.20) methods indicated no causal relationship between EoE and CD. Heterogeneity was not detected (*Q* = 14.5; *p* = 0.070, **Supplementary Figure 2E**). No outlier was identified by MR-PRESSO (*p* = 0.414). Two outliers were screened by MR-Radial (*p* = 0.013). Results of sensitivity analyses after excluding the two outliers became significant (OR, 1.12; 95% CI, 1.02–1.23, **Supplementary Table 3**).

Ten of the screened SNPs were contained in the CD dataset of the IIBDGC consortium (**Supplementary Table 1**). The IVW (OR, 1.06; 95% CI, 0.91–1.23), MR-Egger (OR, 0.95; 95% CI, 0.51–1.76), and weighted median (OR, 0.98; 95% CI, 0.90–1.07) methods tested no causal effect of EoE on CD (**Figure 2F**; **Supplementary Figure 1F**). Heterogeneity existed (*Q* = 63.2; *p* < 0.001, **Supplementary Figure 2F**). The rs56062135 was detected as both a pleiotropic (*p* < 0.001) and heterogenous outlier. The rs1620966 was detected only as a heterogenous outlier by MR-Radial. Results of sensitivity analyses were minimally influenced (**Supplementary Table 3**).

In the meta-analysis, pooled results of IVW (OR, 1.05, 95% CI, 0.96–1.16; *I* ^2 =^ 0), MR-Egger (OR, 1.22, 95% CI, 0.89–1.69; *I* ^2 =^ 0), and weighted median (OR, 1.01, 95% CI, 0.94–1.10; *I* ^2 =^ 26%) confirmed the absence of a casual effect of EoE on CD (**Figure 3G–I**).

### 3.2 Reverse MR analysis

#### 3.2.1 Effect of IBD on EoE

**Figure 4:**
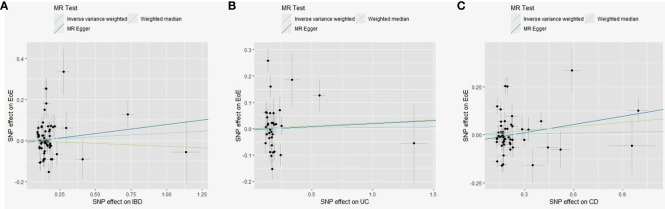
Scatter plots of MR analyses evaluating the causal effect of IBD on EoE **(A)**, UC on EoE **(B)**, and CD on EoE **(C)**. MR, Mendelian randomization; SNP, single-nucleotide polymorphisms; EoE, eosinophilic esophagitis; IBD, inflammatory bowel disease; CD, Crohn’s disease; UC, ulcerative colitis.

A total of 65 SNPs associated with IBD were screened; none has been previously linked to EoE. A total of 59 were available in the outcome dataset (**Supplementary Table 2**). No causal link was found by IVW (OR, 1.04; 95% CI, 0.92–1.16), MR-Egger (OR, 1.09; 95% CI, 0.84–1.42), and weighted median (OR, 0.97; 95% CI, 0.88–1.08) methods (**Supplementary Figure 1F**). Scatter plots are shown in **Figure 4A**. Substantial heterogeneity was detected (*Q* value = 188.4; *p* < 0.001, **Supplementary Figure 3A**). Four pleiotropic outliers were identified by MR-PRESSO (*p* < 0.001) and 12 heterogeneous outliers were identified by MR-Radial. Sensitivity analyses yielded similar results with the primary analysis (**Supplementary Table 3**).

#### 3.2.2 Effect of UC on EoE

A total of 39 SNPs associated with UC were screened; none has been previously linked to EoE. A total of 35 were available in the outcome dataset with one palindromic SNP (rs9891174) further removed. No causal link was found by IVW (OR, 0.98; 95% CI, 0.89–1.08), MR-Egger (OR, 1.02; 95% CI, 0.73–1.43), and weighted median (OR, 1.02; 95% CI, 0.90–1.16) methods (**Figure 4B**; **Supplementary Figure 1G**). Heterogeneity among SNPs was detected (*Q* value = 56.9; *p* = 0.002, **Supplementary Figure 3B**). Three outliers were identified by MR-PRESSO (*p* < 0.001) and nine were identified by MR-Radial. Results of sensitivity analyses remained non-significant (**Supplementary Table 3**).

#### 3.2.3 Effect of CD on EoE

A total of 53 SNPs associated with CD were screened; none has been previously linked to EoE. A total of 52 were available in the EoE database with one palindromic SNP (rs12692254) further removed. No causal link was found by IVW (OR, 1.02; 95% CI, 0.92–1.12), MR-Egger (OR, 1.15; 95% CI, 0.93–1.43), and weighted median (OR, 1.07; 95% CI, 0.99–1.17) methods (**Figure 4C**; **Supplementary Figure 1H**). Significant heterogeneity existed (*Q* value = 192.6; *p* < 0.001, **Supplementary Figure 2C**) and six outliers were identified both by MR-PRESSO (*p* < 0.001) and MR-Radial. Five extra SNPs were revealed only by MR-Radial. Sensitivity analyses confirmed the primary result (**Supplementary Table 3**).

## 4 Discussion

Despite the unneglectable comorbidity rate and shared pathogenesis between EoE and IBD (6), the causal relationship between these two Th cell-mediated GI diseases remains largely unclear. There has always been a dearth of research evaluating the genetic architecture of EoE. Recent publication of EoE GWAS enabled us to use the genetic approach to test for a forward and reverse causal association between EoE and IBD and its subtypes (11). Using publicly available GWAS summary statistics, results of our bidirectional two-sample MR analysis suggested a possible causal association of EoE on IBD, especially on UC (the risk effect is minimal in CD). Genetic liability to IBD or any subtype was not found to correlate with EoE in our analyses.

In MR, genetic markers are employed to determine causality. Genetic variations are independent of illness state and confounders and are therefore unlikely subjected to reverse causation and confounding effect. Differences in outcomes could thus be attributed to exposure. Of note, three assumptions need to be fulfilled in a compelling MR study. First, IVs should be strongly associated with the exposure, which is guaranteed here by the threshold of *p* < 5 × 10^−8^ in the creation of IVs. Second, IVs should influence outcomes through risk factors and not through any direct causal pathway. Third, IVs are not associated with any known or unknown confounders (21). To verify the last two assumptions, we have searched through the PhenoScanner GWAS database to check whether there is a reported relationship between selected SNPs and the outcome as well as its known confounders. The IVs in the forward MR are mainly associated with atopy, which has not been proven to correlate with IBD and its confounding factors like connective tissue disease, infection, antibiotics use, smoking and diet. In addition, IVs utilized in the reverse MR have not been found to be involved in EoE and its known risk factors like food allergy and aeroallergens. Additionally, we attended to the following aspects to enhance the credibility of findings. In source selections, the EoE dataset was obtained from a high-quality GWAS with strict diagnostic criteria (11). The case–control ratio in this study is approximately 1:7, avoiding the bias caused by an extremely unbalanced case–control ratio (22). Moreover, we included two independent IBD GWASs (one with a large sample size and one from the disease-specific consortium with a well-powered case–control design); results obtained by using these two independent datasets were consistent and meta-analyses further strengthened the estimations. Results of the MR study are not free of bias. A primary concern to the validity of results from an MR analysis is pleiotropy, specifically “horizontal pleiotropy”, whereby genetic variants bias the outcome through a bypass without the involvement of exposure. The IVW method, used for our primary analysis, is the most widely used and efficient method but is sensitive to pleiotropy. Several other methods that are more robust to pleiotropy but typically less efficient, such as the MR-Egger, weighted median, and MR-PRESSO methods, are commonly used as sensitivity analyses (21). Here, we performed three kinds of sensitivity analyses based on different emphases of testing and evaluating the pleiotropic effect. The MR-PRESSO method mainly tests horizontal pleiotropy, while MR-Radial tests the contribution of individual IV to Cochran’s *Q* statistic and therefore is more powerful in identifying the source of heterogeneity (17, 19). Generally, consistency in results of primary analyses and most sensitivity analyses (detailed in the results section and **Supplementary Table 3**) supports the robustness of our findings.

Concurrent diagnosis of EoE and IBD has attracted attention clinically, and an exclusion of IBD is recommended when a diagnosis of EoE is made (23). The underlying mechanism remains ambiguous, and several explanations have been proposed. Although involved regions locate at the opposite end of the GI tract, EoE and UC both invoke Th-2-mediated pathways (24, 25) with shared pro-inflammatory cytokines (mainly interleukin-5 and interleukin-13) and shared activation of downstream Janus kinase and signal transducer and activator of transcription (JAK-STAT) pathways (mainly STAT 3 and STAT 6) (26–28). That is to say, an early overactivation of Th-2 immune response in patients with EoE may trigger a subsequent development of UC. In contrast, CD is mainly mediated by Th-1 cellular immune response and thus shows a weaker link with EoE. Besides the adaptive immune response, pathogenesis of both diseases involves upregulation of toll-like receptors, a critical class of proteins in the innate immune system (29, 30). The activation of innate immune response against GI bacteria triggers inflammation in both esophageal and intestinal mucosa. An additional explanation is the impairment of epithelial barrier function and exposure to shared pathogenic environmental factors in both diseases. Elevated interleukin-13 in both diseases downregulate proteins associated with barrier function (desmoglein-1 and filaggrin) and altered epithelial permeability in the GI tract (31–33), permitting interactions between risk environmental factors (food antigens, microbial dysbiosis, antibiotic use, etc.) and the esophageal/intestinal immune system (28, 34, 35). Aberrant immune responses against these antigens provoke mucosal inflammation and cause diseases. Despite the potential explanations above, direct experimental data have not been provided to support the overlap in biological mechanisms of the two diseases. This could be achieved by comparing the inflammatory pattern in the esophageal and intestinal mucosa of patients with co-existing EoE and IBD (6), or in animal models.

Herein, we innovatively conduct a bidirectional two-sample MR study to evaluate the causal relationship between EoE and IBD, providing new insights into understanding the high comorbidity between these two immune-mediated GI diseases. Clinically, the present findings call for an increased awareness of concurrent or subsequent IBD, especially UC, in the management of patients with EoE. Besides regular gastroscopy, colonoscopy might be taken into consideration during the follow-up of patients with EoE. This study inevitably has several limitations. First, as an ethnicity-limited study, whether the findings could be generalized to other ethnic populations remains unclear. With the update of EoE GWAS from other populations, the validation analysis should be conducted to test the robustness of our findings in different populations. Second, the GWAS data of EoE were generated based on children and adolescent patients. Though no evidence of difference in genetic variants between pediatric and adult patients with EoE has been generated, selection bias could not be fully excluded. Third, an absence of detailed information regarding severity stratification, status, and duration of IBD and its subtypes in both FinnGen and IIBDGC GWASs limited the evaluation of selection bias and the performance of further subgroup analyses. Variations in clinical phenotypes, disease severity, and clinical outcome are observed in IBD, and these have been linked to underlying genetic basis (36, 37). Additionally, we preliminarily assessed the genetic overlap between EoE and IBD (data from the ieu-a-31 GWAS dataset) via the linkage disequilibrium score regression (38), and the result showed no evidence of a genetic correlation between the two diseases (genetic correlation = −0.325, *p* = 0.393). Generally, a consistency between results of linkage disequilibrium score regression and MR could make the MR estimates more convincing (39). Therefore, results generated by this study should still be interpreted with caution. Further studies, especially EoE GWASs with a larger sample size, are warranted to resolve the above issues.

## 5 Conclusion

This study leverages support from MR analysis for a potential causal relationship of EoE on IBD (specifically UC). No reverse causal link was revealed. The present findings call for an increased awareness of concurrent or subsequent IBD, especially UC, in the management of patients with EoE. Further GWASs of EoE are needed to confirm our findings, and experimental studies should also be performed to reveal the underlying mechanisms.

## Data availability statement

The original contributions presented in the study are included in the article/**Supplementary Material**. Further inquiries can be directed to the corresponding author.

## Ethics statement

The studies involving humans were approved by Institutional Review Board of the Children’s Hospital of Philadelphia (CHOP). The studies were conducted in accordance with the local legislation and institutional requirements. Written informed consent for participation was not required from the participants or the participants’ legal guardians/next of kin in accordance with the national legislation and institutional requirements.

## Author contributions

RJ: Conceptualization, Data curation, Formal analysis, Investigation, Methodology, Project administration, Software, Validation, Writing – original draft, Writing – review & editing. YZ: Conceptualization, Funding acquisition, Investigation, Resources, Supervision, Validation, Visualization, Writing – review & editing.
