# Supplementary material for: Causal relationship between eosinophilic esophagitis and inflammatory bowel disease: a bidirectional two-sample Mendelian randomization study
Source: Front Immunol. 2024 Apr 24;15:1374107. doi: 10.3389/fimmu.2024.1374107 (PMC11076662; doi:10.3389/fimmu.2024.1374107)
Supplement: Supplementary file 1 [file DataSheet_1.docx]

Table S1 SNPs included in forward MR analyses

| **EoE on IBD (FinnGen)** | | | | | | | | | | | | | | |
| --- | --- | --- | --- | --- | --- | --- | --- | --- | --- | --- | --- | --- | --- | --- |
| **SNP** | **Chr** | **Pos** | **Nearest Gene** | **EA** | **OA** | **EAF** | **Beta. Exposure** | **Se.Exposure** | **Pval.Exposure** | **id.Outcome** | **Outcome** | **Beta.Outcome** | **Se.Outcome** | **Pval.outcome** |
| rs143457388 | 2 | 31402370 | CAPN14 | A | T | 0.0513 | 0.5710 | 0.115 | 2.69×10^-16^ | FinnGen-IBD | IBD | 0.0137 | 0.0344 | 0.6899 |
| rs887992 | 2 | 103524931 | TMEM182 | A | C | 0.6563 | -0.2877 | 0.031 | 4.43×10^-10^ | FinnGen-IBD | IBD | -0.0334 | 0.0155 | 0.0310 |
| rs1438673 | 5 | 110467499 | TSLP/WDR36 | T | C | 0.4983 | -0.3567 | 0.050 | 6.12×10^-22^ | FinnGen-IBD | IBD | -0.0075 | 0.0152 | 0.6199 |
| rs2106984 | 5 | 131953066 | RAD50 | A | T | 0.2031 | 0.2311 | 0.050 | 4.11×10^-08^ | FinnGen-IBD | IBD | 0.0027 | 0.0173 | 0.8750 |
| rs1620996 | 6 | 21602552 | SOX4 | C | T | 0.8915 | -0.3711 | 0.044 | 2.70×10^-08^ | FinnGen-IBD | IBD | 0.0337 | 0.0231 | 0.1450 |
| rs147702004 | 11 | 3865496 | RHOG | T | A | 0.0183 | 0.6678 | 0.180 | 1.15×10^-08^ | FinnGen-IBD | IBD | -0.0001 | 0.0455 | 0.9986 |
| rs61894547 | 11 | 76248630 | EMSY | T | C | 0.0233 | 0.5822 | 0.123 | 4.69×10^-15^ | FinnGen-IBD | IBD | 0.0248 | 0.0392 | 0.5276 |
| rs2279293 | 15 | 61057357 | RORA | G | C | 0.1449 | -0.3711 | 0.037 | 4.66×10^-11^ | FinnGen-IBD | IBD | -0.0564 | 0.0204 | 0.0058 |
| rs56062135 | 15 | 67455630 | SMAD3 | T | C | 0.2303 | 0.2546 | 0.050 | 3.79×10^-10^ | FinnGen-IBD | IBD | 0.0645 | 0.0170 | 0.0001 |
| rs35099084 | 16 | 11189617 | CLEC16A | T | C | 0.1977 | -0.3285 | 0.032 | 1.92×10^-12^ | FinnGen-IBD | IBD | -0.0442 | 0.0199 | 0.0260 |
| **EoE on UC (FinnGen)** | | | | | | | | | | | | | | |
| **SNP** | **Chr** | **Pos** | **Nearest Gene** | **EA** | **OA** | **EAF** | **Beta. Exposure** | **Se.Exposure** | **Pval.Exposure** | **id.Outcome** | **Outcome** | **Beta.Outcome** | **Se.Outcome** | **Pval.outcome** |
| rs143457388 | 2 | 31402370 | CAPN15 | A | T | 0.1300 | -0.0168 | 0.061 | 2.69×10^-17^ | FinnGen-UC | UC | 0.1015 | 0.0425 | 0.0169 |
| rs887992 | 2 | 103524931 | TMEM183 | A | C | 0.1007 | -0.0306 | 0.059 | 4.43×10^-11^ | FinnGen-UC | UC | -0.0303 | 0.0191 | 0.1116 |
| rs1438673 | 5 | 110467499 | TSLP/WDR37 | T | C | 0.0713 | -0.0444 | 0.057 | 6.12×10^-23^ | FinnGen-UC | UC | -0.0038 | 0.0187 | 0.8401 |
| rs2106984 | 5 | 131953066 | RAD51 | A | T | 0.0419 | -0.0583 | 0.055 | 4.11×10^-09^ | FinnGen-UC | UC | 0.0183 | 0.0213 | 0.3907 |
| rs1620996 | 6 | 21602552 | SOX5 | C | T | 0.0126 | -0.0721 | 0.053 | 2.70×10^-09^ | FinnGen-UC | UC | 0.0191 | 0.0285 | 0.5019 |
| rs147702004 | 11 | 3865496 | RHOG | T | A | -0.0168 | -0.0859 | 0.051 | 1.15×10^-09^ | FinnGen-UC | UC | 0.0164 | 0.0560 | 0.7695 |
| rs61894547 | 11 | 76248630 | EMSY | T | C | -0.0461 | -0.0997 | 0.049 | 4.69×10^-16^ | FinnGen-UC | UC | -0.0191 | 0.0483 | 0.6920 |
| rs2279293 | 15 | 61057357 | RORA | G | C | -0.0755 | -0.1135 | 0.047 | 4.66×10^-12^ | FinnGen-UC | UC | -0.0478 | 0.0249 | 0.0545 |
| rs56062135 | 15 | 67455630 | SMAD4 | T | C | -0.1048 | -0.1273 | 0.045 | 3.79×10^-11^ | FinnGen-UC | UC | 0.0639 | 0.0209 | 0.0022 |
| rs35099084 | 16 | 11189617 | CLEC17A | T | C | -0.1342 | -0.1411 | 0.043 | 1.92×10^-13^ | FinnGen-UC | UC | -0.0350 | 0.0244 | 0.1516 |
| **EoE on CD (FinnGen)** | | | | | | | | | | | | | | |
| **SNP** | **Chr** | **Pos** | **Nearest Gene** | **EA** | **OA** | **EAF** | **Beta. Exposure** | **Se.Exposure** | **Pval.Exposure** | **id.Outcome** | **Outcome** | **Beta.Outcome** | **Se.Outcome** | **Pval.outcome** |
| rs143457388 | 2 | 31402370 | CAPN14 | A | T | 0.0513 | 0.5710 | 0.115 | 2.69×10^-16^ | FinnGen-CD | CD | -0.1456 | 0.0722 | 0.0438 |
| rs887992 | 2 | 103524931 | TMEM182 | A | C | 0.6563 | -0.2877 | 0.031 | 4.43×10^-10^ | FinnGen-CD | CD | -0.0629 | 0.0324 | 0.0523 |
| rs1438673 | 5 | 110467499 | TSLP/WDR36 | T | C | 0.4983 | -0.3567 | 0.050 | 6.12×10^-22^ | FinnGen-CD | CD | -0.0053 | 0.0317 | 0.8678 |
| rs2106984 | 5 | 131953066 | RAD50 | A | T | 0.2031 | 0.2311 | 0.050 | 4.11×10^-08^ | FinnGen-CD | CD | -0.0231 | 0.0362 | 0.5232 |
| rs1620996 | 6 | 21602552 | SOX4 | C | T | 0.8915 | -0.3711 | 0.044 | 2.70×10^-08^ | FinnGen-CD | CD | 0.0214 | 0.0485 | 0.6586 |
| rs147702004 | 11 | 3865496 | RHOG | T | A | 0.0183 | 0.6678 | 0.180 | 1.15×10^-08^ | FinnGen-CD | CD | -0.1561 | 0.0952 | 0.1011 |
| rs61894547 | 11 | 76248630 | EMSY | T | C | 0.0233 | 0.5822 | 0.123 | 4.69×10^-15^ | FinnGen-CD | CD | 0.0555 | 0.0816 | 0.4964 |
| rs2279293 | 15 | 61057357 | RORA | G | C | 0.1449 | -0.3711 | 0.037 | 4.66×10^-11^ | FinnGen-CD | CD | -0.0869 | 0.0423 | 0.0400 |
| rs56062135 | 15 | 67455630 | SMAD3 | T | C | 0.2303 | 0.2546 | 0.050 | 3.79×10^-10^ | FinnGen-CD | CD | 0.0814 | 0.0359 | 0.0233 |
| rs35099084 | 16 | 11189617 | CLEC16A | T | C | 0.1977 | -0.3285 | 0.032 | 1.92×10^-12^ | FinnGen-CD | CD | -0.0541 | 0.0414 | 0.1914 |
| **EoE on IBD (IIBDGC)** | | | | | | | | | | | | | | |
| **SNP** | **Chr** | **Pos** | **Nearest Gene** | **EA** | **OA** | **EAF** | **Beta. Exposure** | **Se.Exposure** | **Pval.Exposure** | **id.Outcome** | **Outcome** | **Beta.Outcome** | **Se.Outcome** | **Pval.outcome** |
| rs143457388 | 2 | 31402370 | CAPN16 | A | T | -0.1636 | -0.1549 | 0.041 | 2.69×10^-18^ | ieu-e-31 | IBD | 0.0151 | 0.0406 | 0.7098 |
| rs887992 | 2 | 103524931 | TMEM184 | A | C | -0.1929 | -0.1688 | 0.039 | 4.43×10^-12^ | ieu-e-31 | IBD | 0.0022 | 0.0175 | 0.8989 |
| rs1438673 | 5 | 110467499 | TSLP/WDR38 | T | C | -0.2223 | -0.1826 | 0.037 | 6.12×10^-24^ | ieu-e-31 | IBD | -0.0163 | 0.0168 | 0.3319 |
| rs2106984 | 5 | 131953066 | RAD52 | A | T | -0.2516 | -0.1964 | 0.035 | 4.11×10^-10^ | ieu-e-31 | IBD | -0.0219 | 0.0205 | 0.2870 |
| rs1620996 | 6 | 21602552 | SOX6 | C | T | -0.2810 | -0.2102 | 0.033 | 2.70×10^-10^ | ieu-e-31 | IBD | 0.0370 | 0.0275 | 0.1785 |
| rs147702004 | 11 | 3865496 | RHOG | T | A | -0.3103 | -0.2240 | 0.031 | 1.15×10^-10^ | ieu-e-31 | IBD | 0.0110 | 0.0666 | 0.8694 |
| rs61894547 | 11 | 76248630 | EMSY | T | C | -0.3397 | -0.2378 | 0.029 | 4.69×10^-17^ | ieu-e-31 | IBD | 0.1375 | 0.0417 | 0.0010 |
| rs2279293 | 15 | 61057357 | RORA | G | C | -0.3691 | -0.2516 | 0.027 | 4.66×10^-13^ | ieu-e-31 | IBD | -0.0918 | 0.0247 | 0.0002 |
| rs56062135 | 15 | 67455630 | SMAD5 | T | C | -0.3984 | -0.2655 | 0.025 | 3.79×10^-12^ | ieu-e-31 | IBD | 0.1509 | 0.0198 | <0.0001 |
| rs35099084 | 16 | 11189617 | CLEC18A | T | C | -0.4278 | -0.2793 | 0.023 | 1.92×10^-14^ | ieu-e-31 | IBD | -0.0094 | 0.0205 | 0.6457 |
| **EoE on UC (IIBDGC)** | | | | | | | | | | | | | | |
| **SNP** | **Chr** | **Pos** | **Nearest Gene** | **EA** | **OA** | **EAF** | **Beta. Exposure** | **Se.Exposure** | **Pval.Exposure** | **id.Outcome** | **Outcome** | **Beta.Outcome** | **Se.Outcome** | **Pval.Outcome** |
| rs143457388 | 2 | 31402370 | CAPN16 | A | T | -0.1636 | -0.1549 | 0.041 | 2.69×10^-18^ | ieu-a-32 | UC | 0.0167 | 0.0507 | 0.7426 |
| rs887992 | 2 | 103524931 | TMEM184 | A | C | -0.1929 | -0.1688 | 0.039 | 4.43×10^-12^ | ieu-a-32 | UC | -0.0091 | 0.0221 | 0.6795 |
| rs1438673 | 5 | 110467499 | TSLP/WDR38 | T | C | -0.2223 | -0.1826 | 0.037 | 6.12×10^-24^ | ieu-a-32 | UC | -0.0213 | 0.0213 | 0.3170 |
| rs2106984 | 5 | 131953066 | RAD52 | A | T | -0.2516 | -0.1964 | 0.035 | 4.11×10^-10^ | ieu-a-32 | UC | 0.0006 | 0.0258 | 0.9809 |
| rs1620996 | 6 | 21602552 | SOX6 | C | T | -0.2810 | -0.2102 | 0.033 | 2.70×10^-10^ | ieu-a-32 | UC | 0.0218 | 0.0347 | 0.5296 |
| rs147702004 | 11 | 3865496 | RHOG | T | A | -0.3103 | -0.2240 | 0.031 | 1.15×10^-10^ | ieu-a-32 | UC | 0.0779 | 0.0809 | 0.3354 |
| rs61894547 | 11 | 76248630 | EMSY | T | C | -0.3397 | -0.2378 | 0.029 | 4.69×10^-17^ | ieu-a-32 | UC | 0.1338 | 0.0520 | 0.0101 |
| rs2279293 | 15 | 61057357 | RORA | G | C | -0.3691 | -0.2516 | 0.027 | 4.66×10^-13^ | ieu-a-32 | UC | -0.0774 | 0.0311 | 0.0129 |
| rs56062135 | 15 | 67455630 | SMAD5 | T | C | -0.3984 | -0.2655 | 0.025 | 3.79×10^-12^ | ieu-a-32 | UC | 0.1105 | 0.0248 | 0.0000 |
| rs35099084 | 16 | 11189617 | CLEC18A | T | C | -0.4278 | -0.2793 | 0.023 | 1.92×10^-14^ | ieu-a-32 | UC | -0.0151 | 0.0259 | 0.5602 |
| **EoE on CD (IIBDGC)** | | | | | | | | | | | | | | |
| **SNP** | **Chr** | **Pos** | **Nearest Gene** | **EA** | **OA** | **EAF** | **Beta. Exposure** | **Se.Exposure** | **Pval.Exposure** | **id.Outcome** | **Outcome** | **Beta.Outcome** | **Se.Outcome** | **Pval.outcome** |
| rs143457388 | 2 | 31402370 | CAPN15 | A | T | 0.1300 | -0.0168 | 0.061 | 2.69×10^-17^ | ieu-a-30 | CD | 0.0326 | 0.0562 | 0.5614 |
| rs887992 | 2 | 103524931 | TMEM183 | A | C | 0.1007 | -0.0306 | 0.059 | 4.43×10^-11^ | ieu-a-30 | CD | 0.0214 | 0.0238 | 0.3687 |
| rs1438673 | 5 | 110467499 | TSLP/WDR37 | T | C | 0.0713 | -0.0444 | 0.057 | 6.12×10^-23^ | ieu-a-30 | CD | 0.0021 | 0.0228 | 0.9267 |
| rs2106984 | 5 | 131953066 | RAD51 | A | T | 0.0419 | -0.0583 | 0.055 | 4.11×10^-09^ | ieu-a-30 | CD | -0.0393 | 0.0281 | 0.1622 |
| rs1620996 | 6 | 21602552 | SOX5 | C | T | 0.0126 | -0.0721 | 0.053 | 2.70×10^-09^ | ieu-a-30 | CD | 0.0527 | 0.0375 | 0.1598 |
| rs147702004 | 11 | 3865496 | RHOG | T | A | -0.0168 | -0.0859 | 0.051 | 1.15×10^-09^ | ieu-a-30 | CD | -0.1062 | 0.0945 | 0.2612 |
| rs61894547 | 11 | 76248630 | EMSY | T | C | -0.0461 | -0.0997 | 0.049 | 4.69×10^-16^ | ieu-a-30 | CD | 0.1320 | 0.0573 | 0.0212 |
| rs2279293 | 15 | 61057357 | RORA | G | C | -0.0755 | -0.1135 | 0.047 | 4.66×10^-12^ | ieu-a-30 | CD | -0.0654 | 0.0338 | 0.0531 |
| rs56062135 | 15 | 67455630 | SMAD4 | T | C | -0.1048 | -0.1273 | 0.045 | 3.79×10^-11^ | ieu-a-30 | CD | 0.1931 | 0.0269 | <0.0001 |
| rs35099084 | 16 | 11189617 | CLEC17A | T | C | -0.1342 | -0.1411 | 0.043 | 1.92×10^-13^ | ieu-a-30 | CD | 0.0127 | 0.0275 | 0.6450 |

MR: Mendelian randomization; SNP: single nucleotide polymorphism; Chr: chromosome; Pos: base-pair position; EA: effect allele; OA: other allele; EAF: effect allele frequency. EoE: eosinophilic esophagitis; IBD: inflammatory bowel disease; CD: Crohn’s disease; UC: ulcerative colitis; IIBDGC: International IBD Genetics Consortium.

Table S2 SNPs included in Reverse MR analyses

| **IBD on EoE** | | | | | | | | | | | | | | |
| --- | --- | --- | --- | --- | --- | --- | --- | --- | --- | --- | --- | --- | --- | --- |
| **SNP** | **Chr** | **Pos** | **EA** | **OA** | **id.Exposure** | **Exposure** | **EAF** | **Beta.Exposure** | **Se.Exposure** | **Pval.Exposure** | **Outcome** | **Beta.Outcome** | **Se.Outcome** | **Pval.Outcome** |
| rs10737481 | 1 | 19845021 | G | T | ieu-a-31 | IBD | 0.555 | 0.1411 | 0.0170 | 1.19E-16 | EoE | 0.0108 | 0.0350 | 0.7629 |
| rs10800314 | 1 | 161502999 | A | C | ieu-a-31 | IBD | 0.649 | -0.1431 | 0.0179 | 1.17E-15 | EoE | 0.0894 | 0.0390 | 0.0156 |
| rs11209026 | 1 | 67240275 | A | G | ieu-a-31 | IBD | 0.054 | -0.7263 | 0.0422 | 1.76E-66 | EoE | -0.1263 | 0.0604 | 0.0859 |
| rs1886731 | 1 | 2540642 | C | T | ieu-a-31 | IBD | 0.481 | -0.0971 | 0.0175 | 3.08E-08 | EoE | -0.0185 | 0.0349 | 0.6161 |
| rs3024493 | 1 | 206770623 | A | C | ieu-a-31 | IBD | 0.170 | 0.2130 | 0.0222 | 8.48E-22 | EoE | 0.0701 | 0.0499 | 0.1513 |
| rs35730213 | 1 | 200905101 | C | G | ieu-a-31 | IBD | 0.263 | -0.1514 | 0.0194 | 6.91E-15 | EoE | 0.0123 | 0.0392 | 0.7602 |
| rs7523335 | 1 | 8120150 | A | G | ieu-a-31 | IBD | 0.173 | -0.1405 | 0.0225 | 4.16E-10 | EoE | 0.0629 | 0.0479 | 0.1818 |
| rs112401990 | 2 | 60972192 | A | G | ieu-a-31 | IBD | 0.375 | 0.1422 | 0.0174 | 2.84E-16 | EoE | 0.1488 | 0.0416 | <0.0001 |
| rs112694524 | 2 | 43226582 | A | G | ieu-a-31 | IBD | 0.090 | 0.1883 | 0.0303 | 5.39E-10 | EoE | -0.0907 | 0.0621 | 0.2135 |
| rs11677953 | 2 | 218256940 | A | G | ieu-a-31 | IBD | 0.415 | 0.0976 | 0.0171 | 1.05E-08 | EoE | 0.0605 | 0.0370 | 0.0934 |
| rs2241878 | 2 | 233275072 | C | T | ieu-a-31 | IBD | 0.538 | 0.1480 | 0.0169 | 1.75E-18 | EoE | 0.0455 | 0.0365 | 0.2078 |
| rs4676408 | 2 | 240634984 | A | G | ieu-a-31 | IBD | 0.515 | 0.1181 | 0.0181 | 6.62E-11 | EoE | -0.0160 | 0.0343 | 0.6579 |
| rs4851586 | 2 | 102447804 | C | T | ieu-a-31 | IBD | 0.757 | -0.1224 | 0.0193 | 2.32E-10 | EoE | 0.0097 | 0.0407 | 0.8182 |
| rs1873625 | 3 | 49629531 | A | C | ieu-a-31 | IBD | 0.323 | 0.1773 | 0.0179 | 3.71E-23 | EoE | -0.0212 | 0.0370 | 0.5894 |
| rs45528737 | 4 | 122354400 | T | C | ieu-a-31 | IBD | 0.112 | 0.1668 | 0.0300 | 2.66E-08 | EoE | 0.0695 | 0.0628 | 0.2643 |
| rs6826501 | 4 | 36075054 | T | C | ieu-a-31 | IBD | 0.536 | -0.0928 | 0.0169 | 4.12E-08 | EoE | -0.0401 | 0.0333 | 0.2637 |
| rs10045431 | 5 | 159387525 | C | A | ieu-a-31 | IBD | 0.718 | 0.1774 | 0.0189 | 6.59E-21 | EoE | -0.0343 | 0.0363 | 0.3803 |
| rs13178036 | 5 | 40218427 | C | G | ieu-a-31 | IBD | 0.342 | 0.1009 | 0.0184 | 4.13E-08 | EoE | 0.0202 | 0.0376 | 0.5983 |
| rs17800987 | 5 | 150943866 | G | A | ieu-a-31 | IBD | 0.089 | 0.2017 | 0.0305 | 3.71E-11 | EoE | 0.0656 | 0.0618 | 0.2855 |
| rs254560 | 5 | 135107916 | A | G | ieu-a-31 | IBD | 0.400 | 0.0995 | 0.0171 | 6.16E-09 | EoE | 0.0168 | 0.0358 | 0.6444 |
| rs35260072 | 5 | 132295159 | C | A | ieu-a-31 | IBD | 0.433 | 0.1422 | 0.0170 | 7.07E-17 | EoE | -0.0534 | 0.0332 | 0.1410 |
| rs6873866 | 5 | 96912106 | C | T | ieu-a-31 | IBD | 0.529 | -0.1070 | 0.0176 | 1.09E-09 | EoE | 0.0389 | 0.0362 | 0.2811 |
| rs6880778 | 5 | 40398994 | G | A | ieu-a-31 | IBD | 0.621 | 0.1878 | 0.0173 | 2.14E-27 | EoE | -0.0249 | 0.0344 | 0.4955 |
| rs148844907 | 6 | 31660620 | A | T | ieu-a-31 | IBD | 0.010 | 1.1375 | 0.0963 | 3.63E-32 | EoE | -0.0556 | 0.1489 | 0.7679 |
| rs34190331 | 6 | 111519617 | A | G | ieu-a-31 | IBD | 0.085 | 0.1769 | 0.0303 | 5.39E-09 | EoE | 0.0228 | 0.0649 | 0.7365 |
| rs444210 | 6 | 166976754 | G | A | ieu-a-31 | IBD | 0.546 | 0.1095 | 0.0168 | 7.39E-11 | EoE | -0.0004 | 0.0345 | 0.9918 |
| rs4712528 | 6 | 20678199 | C | G | ieu-a-31 | IBD | 0.788 | 0.1226 | 0.0207 | 3.07E-09 | EoE | 0.0697 | 0.0440 | 0.1031 |
| rs6911490 | 6 | 106074152 | C | T | ieu-a-31 | IBD | 0.779 | -0.1428 | 0.0208 | 6.82E-12 | EoE | 0.0080 | 0.0431 | 0.8579 |
| rs6927172 | 6 | 137681038 | G | C | ieu-a-31 | IBD | 0.214 | 0.1103 | 0.0202 | 4.65E-08 | EoE | -0.1089 | 0.0389 | 0.0164 |
| rs9370774 | 6 | 14721666 | C | T | ieu-a-31 | IBD | 0.201 | -0.1307 | 0.0219 | 2.54E-09 | EoE | -0.0880 | 0.0431 | 0.0750 |
| rs4730272 | 7 | 107837782 | G | A | ieu-a-31 | IBD | 0.513 | -0.1341 | 0.0178 | 4.50E-14 | EoE | -0.0249 | 0.0345 | 0.4967 |
| rs1551399 | 8 | 125527723 | C | A | ieu-a-31 | IBD | 0.616 | 0.1013 | 0.0173 | 5.01E-09 | EoE | 0.0256 | 0.0364 | 0.4854 |
| rs1887428 | 9 | 4984530 | C | G | ieu-a-31 | IBD | 0.619 | -0.1716 | 0.0178 | 6.65E-22 | EoE | 0.1538 | 0.0417 | <0.0001 |
| rs4077515 | 9 | 136372044 | T | C | ieu-a-31 | IBD | 0.424 | 0.1794 | 0.0172 | 1.50E-25 | EoE | 0.0528 | 0.0366 | 0.1416 |
| rs4246905 | 9 | 114790969 | C | T | ieu-a-31 | IBD | 0.730 | 0.1630 | 0.0197 | 1.42E-16 | EoE | -0.0259 | 0.0378 | 0.5204 |
| rs10761659 | 10 | 62685804 | G | A | ieu-a-31 | IBD | 0.554 | 0.1619 | 0.0172 | 4.07E-21 | EoE | -0.0224 | 0.0340 | 0.5332 |
| rs1250573 | 10 | 79282718 | A | G | ieu-a-31 | IBD | 0.296 | -0.1136 | 0.0190 | 2.21E-09 | EoE | 0.1067 | 0.0415 | 0.0059 |
| rs12764283 | 10 | 35241532 | A | G | ieu-a-31 | IBD | 0.337 | 0.1266 | 0.0179 | 1.57E-12 | EoE | 0.0231 | 0.0375 | 0.5436 |
| rs6584283 | 10 | 99530544 | C | T | ieu-a-31 | IBD | 0.511 | -0.1803 | 0.0169 | 1.70E-26 | EoE | 0.0161 | 0.0353 | 0.6538 |
| rs11236797 | 11 | 76588605 | A | C | ieu-a-31 | IBD | 0.470 | 0.1557 | 0.0170 | 4.75E-20 | EoE | 0.2537 | 0.0450 | <0.0001 |
| rs140892874 | 12 | 40430996 | C | T | ieu-a-31 | IBD | 0.027 | 0.4096 | 0.0512 | 1.28E-15 | EoE | -0.0908 | 0.0965 | 0.4430 |
| rs2193041 | 12 | 68108330 | G | A | ieu-a-31 | IBD | 0.389 | 0.1337 | 0.0172 | 6.91E-15 | EoE | -0.1007 | 0.0326 | 0.0071 |
| rs3850378 | 14 | 87951173 | C | T | ieu-a-31 | IBD | 0.096 | 0.1551 | 0.0282 | 3.80E-08 | EoE | 0.1802 | 0.0666 | 0.0022 |
| rs56062135 | 15 | 67163292 | T | C | ieu-a-31 | IBD | 0.237 | 0.1509 | 0.0198 | 2.64E-14 | EoE | 0.2523 | 0.0498 | <0.0001 |
| rs11548656 | 16 | 81883307 | G | A | ieu-a-31 | IBD | 0.034 | -0.2928 | 0.0507 | 7.72E-09 | EoE | -0.0600 | 0.0842 | 0.5416 |
| rs12446550 | 16 | 28532060 | A | G | ieu-a-31 | IBD | 0.410 | 0.1078 | 0.0171 | 2.78E-10 | EoE | -0.0316 | 0.0339 | 0.3835 |
| rs2076756 | 16 | 50722970 | G | A | ieu-a-31 | IBD | 0.271 | 0.1876 | 0.0186 | 5.59E-24 | EoE | 0.0715 | 0.0416 | 0.0756 |
| rs72798422 | 16 | 50833006 | C | T | ieu-a-31 | IBD | 0.042 | 0.2776 | 0.0431 | 1.19E-10 | EoE | 0.3352 | 0.1103 | <0.0001 |
| rs9934775 | 16 | 50349166 | T | C | ieu-a-31 | IBD | 0.161 | -0.1396 | 0.0232 | 1.71E-09 | EoE | 0.0055 | 0.0462 | 0.9096 |
| rs12936409 | 17 | 39887396 | T | C | ieu-a-31 | IBD | 0.476 | 0.1457 | 0.0168 | 3.87E-18 | EoE | -0.0104 | 0.0345 | 0.7729 |
| rs744166 | 17 | 42362183 | G | A | ieu-a-31 | IBD | 0.410 | -0.1207 | 0.0172 | 2.16E-12 | EoE | 0.0373 | 0.0362 | 0.3022 |
| rs2542147 | 18 | 12775852 | T | G | ieu-a-31 | IBD | 0.837 | -0.1513 | 0.0227 | 2.78E-11 | EoE | 0.0021 | 0.0477 | 0.9659 |
| rs10408351 | 19 | 33263138 | A | G | ieu-a-31 | IBD | 0.238 | 0.1378 | 0.0221 | 4.23E-10 | EoE | -0.0913 | 0.0390 | 0.0410 |
| rs142770866 | 19 | 10414696 | A | G | ieu-a-31 | IBD | 0.081 | 0.2300 | 0.0337 | 8.14E-12 | EoE | -0.0667 | 0.0587 | 0.3189 |
| rs6062496 | 20 | 63697746 | A | G | ieu-a-31 | IBD | 0.578 | 0.1650 | 0.0180 | 5.48E-20 | EoE | -0.0040 | 0.0355 | 0.9133 |
| rs1736161 | 21 | 15460903 | A | G | ieu-a-31 | IBD | 0.429 | -0.1233 | 0.0174 | 1.34E-12 | EoE | -0.0656 | 0.0333 | 0.0746 |
| rs2836882 | 21 | 39094644 | A | G | ieu-a-31 | IBD | 0.258 | -0.1963 | 0.0201 | 1.49E-22 | EoE | 0.0919 | 0.0423 | 0.0219 |
| rs1003342 | 22 | 30174033 | G | A | ieu-a-31 | IBD | 0.531 | -0.0950 | 0.0168 | 1.67E-08 | EoE | -0.0299 | 0.0336 | 0.4040 |
| rs7285952 | 22 | 39337091 | G | T | ieu-a-31 | IBD | 0.157 | -0.1760 | 0.0235 | 7.60E-14 | EoE | -0.0442 | 0.0450 | 0.3704 |
| **CD on EoE** | | | | | | | | | | | | | | |
| **SNP** | **Chr** | **Pos** | **EA** | **OA** | **id.Exposure** | **Exposure** | **EAF** | **Beta.Exposure** | **Se.Exposure** | **Pval.Exposure** | **Outcome** | **Beta.Outcome** | **Se.Outcome** | **Pval.Outcome** |
| rs11209026 | 1 | 67240275 | A | G | ieu-a-30 | CD | 0.056 | -0.9952 | 0.0639 | 1.05E-54 | EoE | -0.1263 | 0.0604 | 0.0859 |
| rs3024505 | 1 | 206766559 | A | G | ieu-a-30 | CD | 0.162 | 0.1779 | 0.0302 | 3.90E-09 | EoE | 0.0703 | 0.0499 | 0.1492 |
| rs6588243 | 1 | 67137700 | C | A | ieu-a-30 | CD | 0.590 | 0.1317 | 0.0234 | 1.78E-08 | EoE | -0.1368 | 0.0309 | 0.0002 |
| rs6704109 | 1 | 172887910 | T | C | ieu-a-30 | CD | 0.256 | 0.2020 | 0.0256 | 2.77E-15 | EoE | -0.0114 | 0.0392 | 0.7818 |
| rs697693 | 1 | 7826364 | A | G | ieu-a-30 | CD | 0.201 | 0.1723 | 0.0281 | 8.36E-10 | EoE | -0.0329 | 0.0422 | 0.4710 |
| rs7543234 | 1 | 155283517 | T | C | ieu-a-30 | CD | 0.239 | 0.1555 | 0.0267 | 6.10E-09 | EoE | 0.0464 | 0.0411 | 0.2559 |
| rs112401990 | 2 | 60972192 | A | G | ieu-a-30 | CD | 0.373 | 0.1322 | 0.0237 | 2.35E-08 | EoE | 0.1488 | 0.0416 | <0.0001 |
| rs12692254 | 2 | 233252565 | T | A | ieu-a-30 | CD | 0.543 | 0.3014 | 0.0232 | 1.86E-38 | EoE | 0.0373 | 0.0363 | 0.3035 |
| rs4851586 | 2 | 102447804 | C | T | ieu-a-30 | CD | 0.760 | -0.1689 | 0.0261 | 9.94E-11 | EoE | 0.0097 | 0.0407 | 0.8182 |
| rs78487399 | 2 | 43582208 | C | G | ieu-a-30 | CD | 0.101 | 0.2259 | 0.0370 | 1.03E-09 | EoE | -0.0659 | 0.0540 | 0.2820 |
| rs1873625 | 3 | 49629531 | A | C | ieu-a-30 | CD | 0.320 | 0.1807 | 0.0243 | 1.09E-13 | EoE | -0.0212 | 0.0370 | 0.5894 |
| rs13135092 | 4 | 102276925 | G | A | ieu-a-30 | CD | 0.095 | 0.2215 | 0.0389 | 1.21E-08 | EoE | 0.0966 | 0.0620 | 0.1056 |
| rs12717899 | 5 | 142102776 | T | G | ieu-a-30 | CD | 0.794 | 0.1592 | 0.0289 | 3.59E-08 | EoE | -0.1594 | 0.0372 | 0.0005 |
| rs147018773 | 5 | 150858959 | T | C | ieu-a-30 | CD | 0.097 | 0.3217 | 0.0375 | 8.89E-18 | EoE | 0.0273 | 0.0609 | 0.6645 |
| rs2188962 | 5 | 132435113 | T | C | ieu-a-30 | CD | 0.440 | 0.2124 | 0.0228 | 1.36E-20 | EoE | -0.0764 | 0.0325 | 0.0354 |
| rs4921497 | 5 | 159421245 | G | C | ieu-a-30 | CD | 0.329 | 0.1603 | 0.0244 | 5.49E-11 | EoE | -0.0555 | 0.0354 | 0.1537 |
| rs6873866 | 5 | 96912106 | C | T | ieu-a-30 | CD | 0.535 | -0.1681 | 0.0239 | 2.06E-12 | EoE | 0.0389 | 0.0362 | 0.2811 |
| rs7713270 | 5 | 40439961 | T | C | ieu-a-30 | CD | 0.624 | 0.2966 | 0.0241 | 6.97E-35 | EoE | -0.0250 | 0.0345 | 0.4951 |
| rs114607072 | 6 | 31384163 | T | G | ieu-a-30 | CD | 0.040 | 0.4418 | 0.0629 | 2.20E-12 | EoE | -0.0655 | 0.0817 | 0.4938 |
| rs12194825 | 6 | 20835029 | A | T | ieu-a-30 | CD | 0.186 | -0.1719 | 0.0298 | 8.00E-09 | EoE | 0.0502 | 0.0452 | 0.2644 |
| rs140054334 | 6 | 31619265 | T | C | ieu-a-30 | CD | 0.038 | 0.3498 | 0.0628 | 2.57E-08 | EoE | -0.1582 | 0.0884 | 0.1720 |
| rs148844907 | 6 | 31660620 | A | T | ieu-a-30 | CD | 0.008 | 0.9580 | 0.1419 | 1.47E-11 | EoE | -0.0556 | 0.1489 | 0.7679 |
| rs28701841 | 6 | 106082455 | A | G | ieu-a-30 | CD | 0.117 | 0.2243 | 0.0373 | 1.85E-09 | EoE | -0.0261 | 0.0546 | 0.6603 |
| rs444210 | 6 | 166976754 | G | A | ieu-a-30 | CD | 0.547 | 0.1634 | 0.0229 | 1.02E-12 | EoE | -0.0004 | 0.0345 | 0.9918 |
| rs1456896 | 7 | 50264865 | T | C | ieu-a-30 | CD | 0.698 | 0.1393 | 0.0251 | 2.90E-08 | EoE | 0.0119 | 0.0374 | 0.7566 |
| rs921720 | 8 | 125522429 | G | A | ieu-a-30 | CD | 0.619 | 0.1629 | 0.0237 | 6.40E-12 | EoE | 0.0114 | 0.0359 | 0.7573 |
| rs1887428 | 9 | 4984530 | C | G | ieu-a-30 | CD | 0.623 | -0.1681 | 0.0243 | 4.22E-12 | EoE | 0.1538 | 0.0417 | <0.0001 |
| rs3810936 | 9 | 114790605 | C | T | ieu-a-30 | CD | 0.698 | 0.2078 | 0.0263 | 2.46E-15 | EoE | -0.0416 | 0.0363 | 0.2899 |
| rs4077515 | 9 | 136372044 | T | C | ieu-a-30 | CD | 0.420 | 0.2159 | 0.0235 | 4.37E-20 | EoE | 0.0528 | 0.0366 | 0.1416 |
| rs10761659 | 10 | 62685804 | G | A | ieu-a-30 | CD | 0.553 | 0.2120 | 0.0237 | 3.42E-19 | EoE | -0.0224 | 0.0340 | 0.5332 |
| rs1250573 | 10 | 79282718 | A | G | ieu-a-30 | CD | 0.287 | -0.1709 | 0.0264 | 9.01E-11 | EoE | 0.1067 | 0.0415 | 0.0059 |
| rs1332099 | 10 | 99538694 | C | T | ieu-a-30 | CD | 0.515 | -0.2116 | 0.0231 | 4.36E-20 | EoE | 0.0478 | 0.0364 | 0.1834 |
| rs2505640 | 10 | 35170569 | G | A | ieu-a-30 | CD | 0.644 | -0.1457 | 0.0237 | 7.61E-10 | EoE | 0.0096 | 0.0365 | 0.7986 |
| rs11236797 | 11 | 76588605 | A | C | ieu-a-30 | CD | 0.473 | 0.1811 | 0.0231 | 4.85E-15 | EoE | 0.2537 | 0.0450 | <0.0001 |
| rs11564236 | 12 | 40434504 | T | A | ieu-a-30 | CD | 0.034 | 0.5191 | 0.0595 | 2.85E-18 | EoE | -0.0748 | 0.0972 | 0.5235 |
| rs1932990 | 13 | 43886106 | T | C | ieu-a-30 | CD | 0.254 | 0.1529 | 0.0263 | 6.02E-09 | EoE | 0.1335 | 0.0447 | 0.0010 |
| rs4902642 | 14 | 68743482 | A | G | ieu-a-30 | CD | 0.409 | -0.1292 | 0.0236 | 4.34E-08 | EoE | 0.0199 | 0.0357 | 0.5829 |
| rs56062135 | 15 | 67163292 | T | C | ieu-a-30 | CD | 0.234 | 0.1931 | 0.0269 | 7.45E-13 | EoE | 0.2523 | 0.0498 | <0.0001 |
| rs147684209 | 16 | 28855740 | C | T | ieu-a-30 | CD | 0.369 | 0.1549 | 0.0244 | 2.34E-10 | EoE | 0.0139 | 0.0360 | 0.7068 |
| rs2076756 | 16 | 50722970 | G | A | ieu-a-30 | CD | 0.284 | 0.3998 | 0.0242 | 3.24E-61 | EoE | 0.0715 | 0.0416 | 0.0756 |
| rs72798422 | 16 | 50833006 | C | T | ieu-a-30 | CD | 0.048 | 0.5904 | 0.0508 | 3.19E-31 | EoE | 0.3352 | 0.1103 | <0.0001 |
| rs3091315 | 17 | 34266646 | G | A | ieu-a-30 | CD | 0.266 | -0.1795 | 0.0263 | 9.52E-12 | EoE | -0.0324 | 0.0378 | 0.4254 |
| rs744166 | 17 | 42362183 | G | A | ieu-a-30 | CD | 0.408 | -0.1293 | 0.0233 | 2.92E-08 | EoE | 0.0373 | 0.0362 | 0.3022 |
| rs907092 | 17 | 39766006 | A | G | ieu-a-30 | CD | 0.471 | 0.1304 | 0.0228 | 1.01E-08 | EoE | -0.0163 | 0.0344 | 0.6525 |
| rs80262450 | 18 | 12818923 | A | G | ieu-a-30 | CD | 0.113 | 0.2831 | 0.0353 | 1.08E-15 | EoE | 0.0295 | 0.0559 | 0.6072 |
| rs2129944 | 19 | 10405522 | G | T | ieu-a-30 | CD | 0.291 | -0.1562 | 0.0271 | 7.81E-09 | EoE | 0.0067 | 0.0390 | 0.8677 |
| rs281379 | 19 | 48711017 | A | G | ieu-a-30 | CD | 0.489 | 0.1398 | 0.0238 | 4.26E-09 | EoE | -0.0049 | 0.0349 | 0.8925 |
| rs8178977 | 19 | 1106478 | C | G | ieu-a-30 | CD | 0.239 | 0.1928 | 0.0274 | 2.06E-12 | EoE | 0.0317 | 0.0426 | 0.4618 |
| rs1056441 | 20 | 63738996 | C | T | ieu-a-30 | CD | 0.698 | 0.1670 | 0.0255 | 5.44E-11 | EoE | 0.0142 | 0.0385 | 0.7187 |
| rs1297271 | 21 | 15450844 | T | C | ieu-a-30 | CD | 0.430 | -0.1549 | 0.0237 | 6.28E-11 | EoE | -0.0694 | 0.0331 | 0.0591 |
| rs151314883 | 22 | 39339082 | A | G | ieu-a-30 | CD | 0.158 | -0.2240 | 0.0327 | 7.12E-12 | EoE | -0.0451 | 0.0452 | 0.3628 |
| rs8137950 | 22 | 21615351 | C | T | ieu-a-30 | CD | 0.200 | 0.1740 | 0.0286 | 1.17E-09 | EoE | -0.0207 | 0.0436 | 0.6571 |
| **UC on EoE** | | | | | | | | | | | | | | |
| **SNP** | **Chr** | **Pos** | **EA** | **OA** | **id.Exposure** | **Exposure** | **EAF** | **Beta.Exposure** | **Se.Exposure** | **Pval.Exposure** | **Outcome** | **Beta.Outcome** | **Se.Outcome** | **Pval.Outcome** |
| rs10737481 | 1 | 19845021 | G | T | ieu-a-32 | UC | 0.556 | 0.2501 | 0.0216 | 4.37E-31 | EoE | 0.0108 | 0.5565 | 0.7629 |
| rs10917545 | 1 | 19801684 | A | G | ieu-a-32 | UC | 0.882 | -0.1851 | 0.0335 | 3.29E-08 | EoE | -0.0591 | 0.8822 | 0.3361 |
| rs11209026 | 1 | 67240275 | A | G | ieu-a-32 | UC | 0.059 | -0.5617 | 0.0517 | 1.58E-27 | EoE | -0.1263 | 0.0589 | 0.0859 |
| rs1801274 | 1 | 161509955 | G | A | ieu-a-32 | UC | 0.483 | -0.1829 | 0.0217 | 3.78E-17 | EoE | 0.0899 | 0.4831 | 0.0123 |
| rs1886731 | 1 | 2540642 | C | T | ieu-a-32 | UC | 0.481 | -0.1405 | 0.0221 | 2.25E-10 | EoE | -0.0185 | 0.4806 | 0.6161 |
| rs3024493 | 1 | 206770623 | A | C | ieu-a-32 | UC | 0.168 | 0.2363 | 0.0276 | 1.09E-17 | EoE | 0.0701 | 0.1681 | 0.1513 |
| rs35730213 | 1 | 200905101 | C | G | ieu-a-32 | UC | 0.267 | -0.1670 | 0.0245 | 8.81E-12 | EoE | 0.0123 | 0.2666 | 0.7602 |
| rs7523335 | 1 | 8120150 | A | G | ieu-a-32 | UC | 0.177 | -0.1704 | 0.0285 | 2.29E-09 | EoE | 0.0629 | 0.1767 | 0.1818 |
| rs10182512 | 2 | 60962334 | A | G | ieu-a-32 | UC | 0.350 | 0.1608 | 0.0223 | 5.19E-13 | EoE | 0.1604 | 0.3499 | <0.0001 |
| rs12612675 | 2 | 218268414 | G | A | ieu-a-32 | UC | 0.403 | 0.1229 | 0.0219 | 1.98E-08 | EoE | 0.0618 | 0.4028 | 0.0868 |
| rs4676410 | 2 | 240624322 | A | G | ieu-a-32 | UC | 0.197 | 0.2078 | 0.0284 | 2.46E-13 | EoE | 0.0172 | 0.1971 | 0.7035 |
| rs9823546 | 3 | 49668079 | A | T | ieu-a-32 | UC | 0.310 | 0.1769 | 0.0223 | 2.29E-15 | EoE | -0.0208 | 0.3104 | 0.5985 |
| rs114152040 | 5 | 40444884 | A | G | ieu-a-32 | UC | 0.032 | 0.3396 | 0.0623 | 4.95E-08 | EoE | 0.1864 | 0.0317 | 0.0450 |
| rs254559 | 5 | 135109292 | A | C | ieu-a-32 | UC | 0.404 | 0.1243 | 0.0215 | 7.63E-09 | EoE | 0.0067 | 0.4041 | 0.8546 |
| rs56167332 | 5 | 159400761 | A | C | ieu-a-32 | UC | 0.342 | 0.1516 | 0.0231 | 5.30E-11 | EoE | -0.0353 | 0.3416 | 0.3742 |
| rs148844907 | 6 | 31660620 | A | T | ieu-a-32 | UC | 0.010 | 1.3413 | 0.1089 | 7.17E-35 | EoE | -0.0556 | 0.0102 | 0.7679 |
| rs6933404 | 6 | 137638098 | C | T | ieu-a-32 | UC | 0.216 | 0.1668 | 0.0252 | 3.68E-11 | EoE | -0.1042 | 0.2156 | 0.0211 |
| rs7752873 | 6 | 106131457 | T | C | ieu-a-32 | UC | 0.137 | 0.1823 | 0.0303 | 1.83E-09 | EoE | 0.0212 | 0.1368 | 0.6824 |
| rs10272963 | 7 | 107846457 | T | C | ieu-a-32 | UC | 0.426 | -0.1719 | 0.0216 | 1.69E-15 | EoE | -0.0217 | 0.4261 | 0.5461 |
| rs798502 | 7 | 2750246 | C | A | ieu-a-32 | UC | 0.283 | -0.1365 | 0.0239 | 1.21E-08 | EoE | -0.0146 | 0.2831 | 0.7128 |
| rs989960 | 7 | 107805282 | T | C | ieu-a-32 | UC | 0.425 | -0.1291 | 0.0215 | 1.77E-09 | EoE | 0.0215 | 0.4249 | 0.5551 |
| rs1887428 | 9 | 4984530 | C | G | ieu-a-32 | UC | 0.623 | -0.1767 | 0.0224 | 3.36E-15 | EoE | 0.1538 | 0.6229 | <0.0001 |
| rs3829111 | 9 | 136375031 | A | G | ieu-a-32 | UC | 0.417 | 0.1563 | 0.0214 | 2.89E-13 | EoE | 0.0592 | 0.4167 | 0.1020 |
| rs4574921 | 9 | 114776054 | T | C | ieu-a-32 | UC | 0.741 | 0.1506 | 0.0256 | 4.24E-09 | EoE | -0.0168 | 0.7408 | 0.6848 |
| rs7911680 | 10 | 99533711 | C | A | ieu-a-32 | UC | 0.490 | -0.1718 | 0.0213 | 8.27E-16 | EoE | 0.0333 | 0.4901 | 0.4651 |
| rs2212434 | 11 | 76570549 | T | C | ieu-a-32 | UC | 0.460 | 0.1419 | 0.0213 | 2.46E-11 | EoE | 0.2579 | 0.4596 | <0.0001 |
| rs483905 | 11 | 96290263 | A | G | ieu-a-32 | UC | 0.294 | 0.1289 | 0.0228 | 1.57E-08 | EoE | 0.0601 | 0.2943 | 0.1248 |
| rs484356 | 11 | 114535917 | G | C | ieu-a-32 | UC | 0.328 | -0.1342 | 0.0228 | 3.95E-09 | EoE | -0.0422 | 0.3277 | 0.2695 |
| rs12817473 | 12 | 68103628 | G | A | ieu-a-32 | UC | 0.382 | 0.1907 | 0.0217 | 1.71E-18 | EoE | -0.0878 | 0.3822 | 0.0192 |
| rs1359946 | 13 | 26962835 | A | G | ieu-a-32 | UC | 0.196 | 0.1583 | 0.0269 | 3.84E-09 | EoE | -0.0891 | 0.1961 | 0.0609 |
| rs9891174 | 17 | 39875549 | A | T | ieu-a-32 | UC | 0.470 | 0.1452 | 0.0212 | 7.17E-12 | EoE | -0.0057 | 0.4704 | 0.8747 |
| rs6017342 | 20 | 44436388 | C | A | ieu-a-32 | UC | 0.538 | 0.1913 | 0.0240 | 1.38E-15 | EoE | 0.0152 | 0.5377 | 0.6863 |
| rs6062496 | 20 | 63697746 | A | G | ieu-a-32 | UC | 0.573 | 0.1585 | 0.0224 | 1.47E-12 | EoE | -0.0040 | 0.5726 | 0.9133 |
| rs9977672 | 21 | 39091357 | A | G | ieu-a-32 | UC | 0.251 | -0.2450 | 0.0261 | 6.21E-21 | EoE | 0.1000 | 0.2513 | 0.0142 |
| rs137845 | 22 | 50001001 | G | A | ieu-a-32 | UC | 0.515 | 0.1182 | 0.0212 | 2.38E-08 | EoE | -0.0558 | 0.5149 | 0.2255 |

MR: Mendelian randomization; SNP: single nucleotide polymorphism; Chr: chromosome; Pos: base-pair position; EA: effect allele; OA: other allele; EAF: effect allele frequency. EoE: eosinophilic esophagitis; IBD: inflammatory bowel disease; CD: Crohn’s disease; UC: ulcerative colitis.

**Table S3 Sensitivity analyses results**

|  | | Primary result,  OR [95% CI] | SA-A,  OR [95% CI] | SNPs excluded in SA-A | SA-B,  OR [95% CI] | SNPs excluded in SA-B | SA-C,  OR [95% CI] |
| --- | --- | --- | --- | --- | --- | --- | --- |
| **Forward MR analyses** | | | | | | | |
| EoE on IBD | FinnGen | 1.07 [1.02, 1.13] | 1.05 [1.00-1.10] | rs56062135 | 1.07 [1.02-1.11] | rs1620996, rs56062135 | 1.07 [1.02-1.11] |
|  | IIBDGC | 1.10 [0.98-1.23] | 1.05 [0.94-1.14] | rs56062135 | 1.08 [0.93-1.25] | rs2279293, rs61894547, rs1620996 | 1.01 [0.96-1.07] |
| EoE on UC | FinnGen | 1.08 [1.02, 1.14] | NA | NA | NA | NA | NA |
|  | IIBDGC | 1.11 [1.02-1.20] | 1.08 [1.02-1.14] | rs56062135 | 1.08 [1.02-1.14] | rs56062135 | 1.08 [1.02-1.14] |
| EoE on CD | FinnGen | 1.05 [0.94, 1.18] | NA | NA | 1.12 [1.02, 1.23] | rs147702004, rs143457388 | 1.12 [1.02, 1.23] |
|  | IIBDGC | 1.06 [0.91-1.23] | 1.00 [0.92-1.09] | rs56062135 | 1.01 [0.93-1.11] | rs1620966 | 1.01 [0.93-1.11] |
| **Reverse MR analyses** | | | | | | | |
| IBD on EoE | | 1.04 [0.92-1.16] | 0.99 [0.90-1.09] | rs11236797, rs112401990, rs1873625, rs56062135 | 1.05 [0.98-1.13] | rs11236797, rs112401990, rs56062135, rs72798422, rs3850378, rs2836882, rs10800314, rs10408351, rs1250573, rs6927172, rs2193041, rs1887428 | 1.06 [0.99-1.14] |
| UC on EoE | | 0.98 [0.89-1.08] | 1.02 [0.95-1.09] | rs10182512, rs1887428, rs2212434 | 1.01 [0.95-1.07] | rs10182512, rs1887428, rs2212434, rs12817473, rs1359946, rs9977672, rs1801274, rs6933404, rs11209026 | 1.01 [0.95-1.07] |
| CD on EoE | | 1.02 [0.92-1.12] | 0.98 [0.89-1.08] | rs11236797, rs112401990, rs12717899, rs1887428, rs1932990, rs56062135, rs6588243 | 1.05 [0.97-1.15] | rs11236797, rs112401990, rs12717899, rs1887428, rs1932990, rs56062135,  rs72798422, rs1932900, rs1297271, rs2188962, rs1250573, rs6588243 | 1.05 [0.97-1.15] |

SA-A: sensitivity analysis A, which removed pleiotropic outliers detected by the MR-PRESSO method SA-B: sensitivity analysis B, which removed heterogenous outliers detected by the MR-Radial method. SA-C: sensitivity analysis A, which removed outliers detected by either methods. MR: Mendelian randomization; SNP: single nucleotide polymorphism; EoE: eosinophilic esophagitis; IBD: inflammatory bowel disease; CD: Crohn’s disease; UC: ulcerative colitis. OR: odds ratio; CI: confidence interval. ; IIBDGC: International IBD Genetics Consortium.


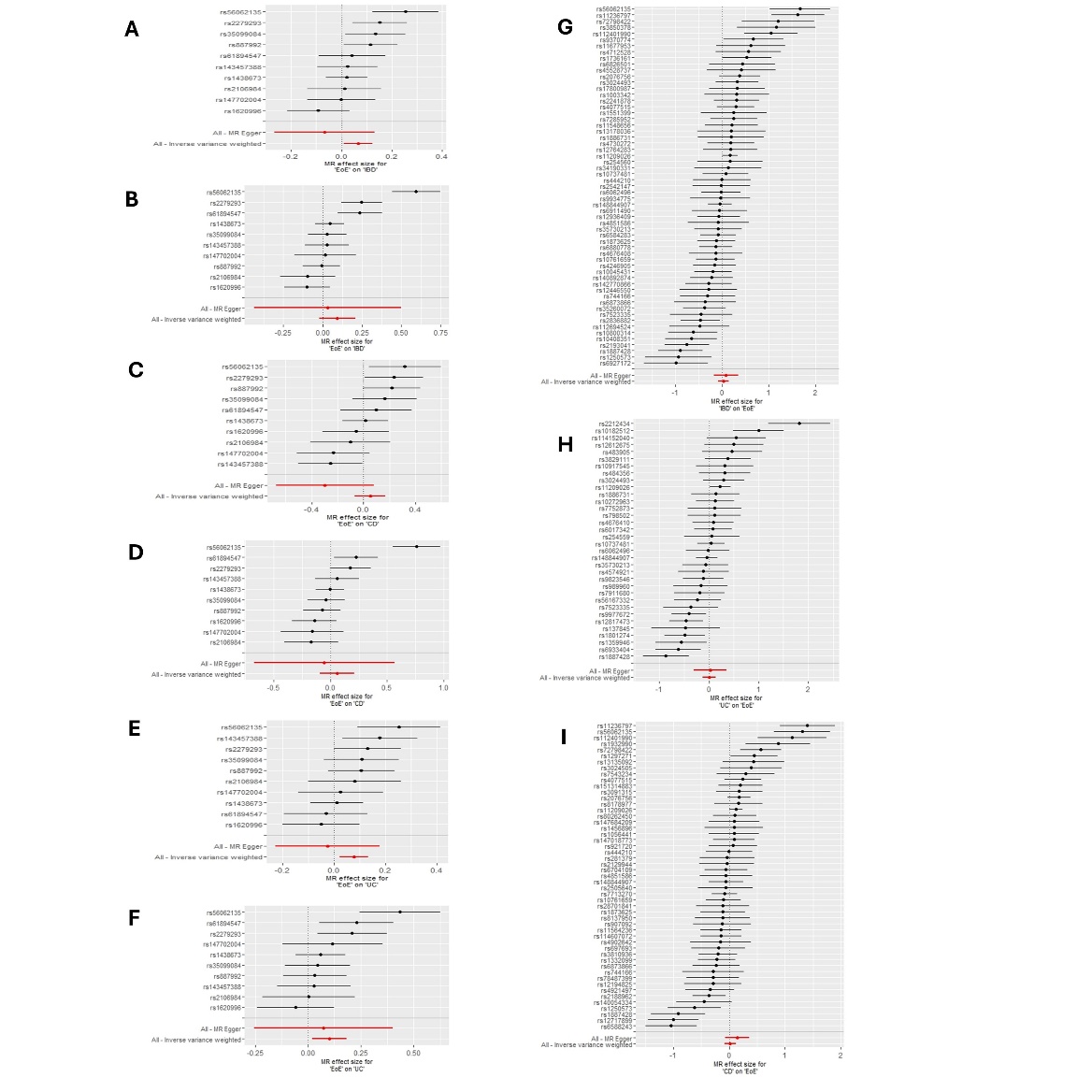


**Figure S1.** Effect size of SNPs for EoE on IBD based on outcome database of FinnGen (A) and IIBDGC (B), EoE on CD based on outcome database of FinnGen (C) and IIBDGC (D), EoE on UC based on outcome database of FinnGen (E) and IIBDGC (F) as well as IBD on EoE (G), UC on EoE (H) and CD on EoE (I). SNP: single nucleotide polymorphisms; EoE: eosinophilic esophagitis; IBD: inflammatory bowel disease; CD: Crohn’s disease; UC: ulcerative colitis; IIBDGC: International IBD Genetics Consortium.


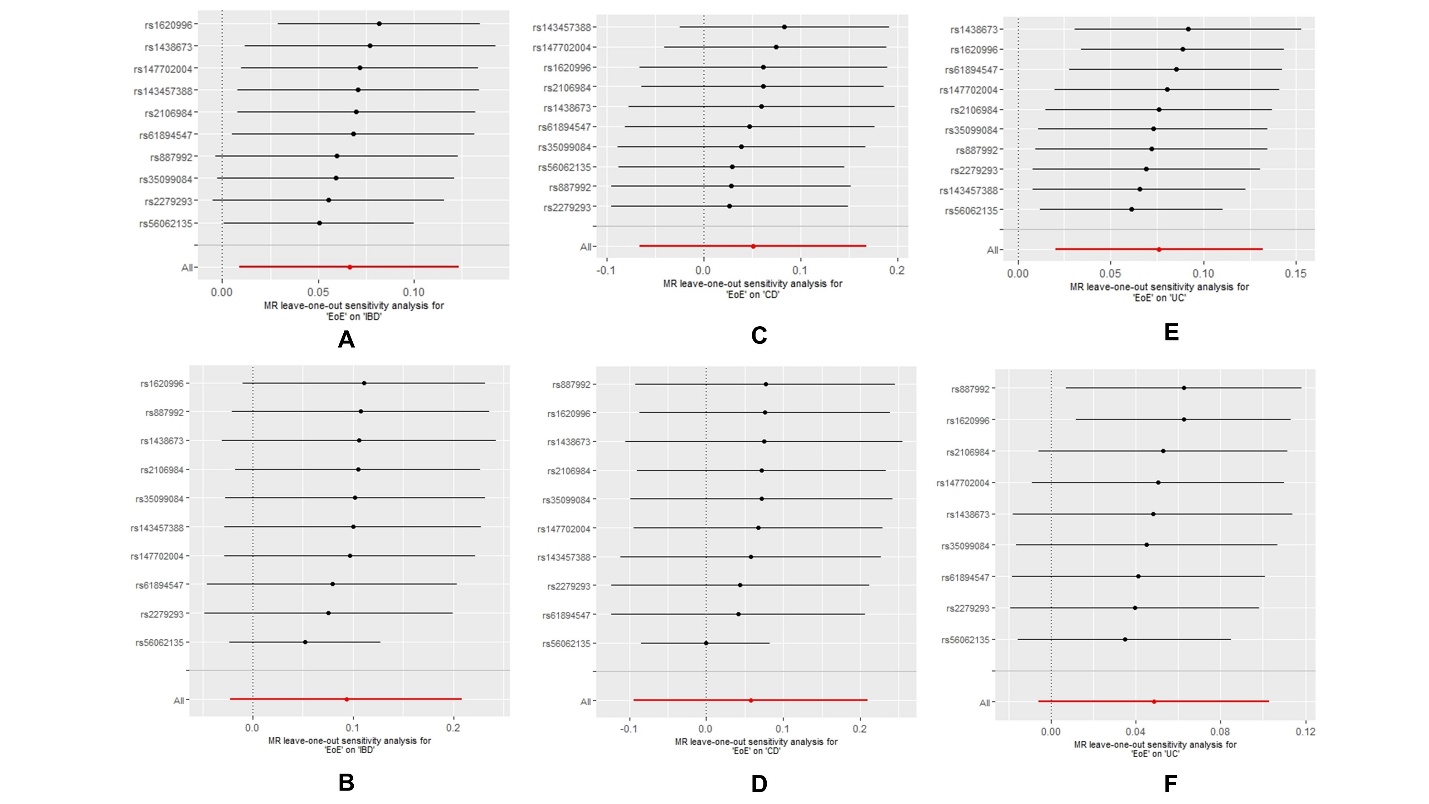


**Figure S2**. Results of leave-one-out sensitivity analyses for EoE on IBD based on outcome database of FinnGen (A) and IIBDGC (B), EoE on CD based on outcome database of FinnGen (C) and IIBDGC (D), EoE on UC based on outcome database of FinnGen(E) and IIBDGC (F). MR: Mendelian randomization; EoE: eosinophilic esophagitis; IBD: inflammatory bowel disease; CD: Crohn’s disease; UC: ulcerative colitis; IIBDGC: International IBD Genetics Consortium.


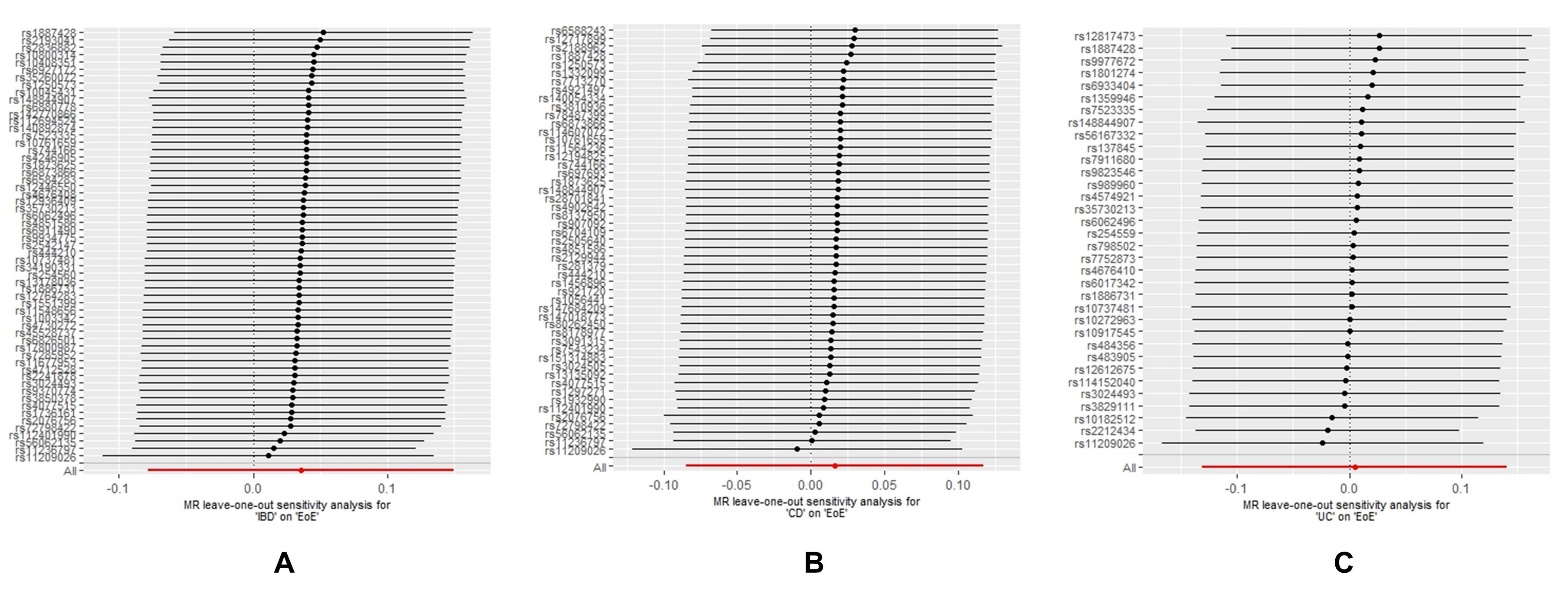


**Figure S3.** Results of leave-one-out sensitivity analyses for IBD on EoE (A), UC on EoE (B) and CD on EoE (C). MR: Mendelian randomization; EoE: eosinophilic esophagitis; IBD: inflammatory bowel disease; CD: Crohn’s disease; UC: ulcerative colitis.
