# Supplementary material for: Causal relationship between eosinophilic esophagitis and inflammatory bowel disease: a bidirectional two-sample Mendelian randomization study
Source: Front Immunol. 2024 Apr 24;15:1374107. doi: 10.3389/fimmu.2024.1374107 (PMC11076662; doi:10.3389/fimmu.2024.1374107)
Supplement: Supplementary file 2 [file DataSheet_2.docx]

**STROBE-MR checklist of recommended items to address in reports of Mendelian randomization studies**^1^ ^2^

| **Item No.** | **Section** | **Checklist item** | **Relevant text from manuscript** |
| --- | --- | --- | --- |
| 1 | **TITLE and ABSTRACT** | Indicate Mendelian randomization (MR) as the study’s design in the title and/or the abstract if that is a main purpose of the study | Title: Causal Relationship between Eosinophilic Esophagitis and Inflammatory Bowel Disease: A Bidirectional Two-Sample Mendelian Randomization Study.  Abstract: We investigated the causal relationship between EoE and IBD and its subtypes via a two-sample bidirectional Mendelian randomization (MR) approach. |
|  | **INTRODUCTION** |  |  |
| 2 | **Background** | Explain the scientific background and rationale for the reported study. What is the exposure? Is a potential causal relationship between exposure and outcome plausible? Justify why MR is a helpful method to address the study question | Exposure: Eosinophilic esophagitis (EoE) is a T helper (Th) type 2 cell immune-mediated upper gastrointestinal (GI) disease.  Potential causal relationship: A large-scale prospective cohort study reported that the prevalence of subsequent EoE on primary IBD and subsequent IBD on primary EoE were 980 and 3,322 per 10,0000 persons, respectively [6]. The high comorbidity rate is speculated to be due to an overlap in pathogenic mechanisms of the two diseases [7, 8]  Mendelian randomization (MR) is an approach that uses the unique properties of genotype to investigate causal relationships, which offers the advantage of minimizing bias caused by confounding factors and reverse causality [9, 10]. |
| 3 | **Objectives** | State specific objectives clearly, including pre-specified causal hypotheses (if any). State that MR is a method that, under specific assumptions, intends to estimate causal effects | With the publishment of a well powered genome-wide association study (GWAS) of EoE in 2022[11], we performed a bidirectional MR study to investigate the causal relationship between EoE and IBD and its subtypes (ulcerative colitis [UC] and Crohn’s disease [CD]). |
|  | **METHODS** |  |  |
| 4 | **Study design and data sources** | Present key elements of the study design early in the article. Consider including a table listing sources of data for all phases of the study. For each data source contributing to the analysis, describe the following: |  |
|  | a) | Setting: Describe the study design and the underlying population, if possible. Describe the setting, locations, and relevant dates, including periods of recruitment, exposure, follow-up, and data collection, when available. | The EoE dataset originated from a meta-analysis of GWAS (ID GCST90027899) [11]. The open-source meta-analysis included 1,930 patients with EoE and 13,634 controls of European ancestry. The GWAS summary data of IBD and its subtypes were obtained from the latest FinnGen datasets (released on Dec 8, 2023, GWAS IDs: finngen_R10_K11_IBD_STRICT, finngen_R10_K11_CD_STRICT2 and finngen_R10_K11_UC_STRICT2) which contain 9,083 IBD, 2,033 CD and 5,931 UC patients of European ancestry, respectively. Detailed information regarding definition of cases, genotype platforms and statistical analysis protocols are available at the FinnGen website [https:/www.finngen.fi/en/]. For validation, we further included three GWAS summary datasets (ieu-a-30, ieu-a-31 and ieu-a-32) published by the International IBD Genetics Consortium (IIBDGC) which contain 5,956 CD, 12,882 IBD and 6,968 UC patients of European ancestry, respectively [12] |
|  | b) | Participants: Give the eligibility criteria, and the sources and methods of selection of participants. Report the sample size, and whether any power or sample size calculations were carried out prior to the main analysis | The diagnosis of EoE was both clinically and pathologically confirmed.  Detailed information regarding definition of cases, genotype platforms and statistical analysis protocols are available at the FinnGen website [https:/www.finngen.fi/en/]. |
|  | c) | Describe measurement, quality control and selection of genetic variants | The significant threshold was set as P < 5 ×10^-8^ to filter single nucleotide polymorphisms (SNPs) strongly correlated with the exposure. We further performed a linkage disequilibrium clumping and excluded SNPs with r2 ≥0.001 and clump distance ≤ 10,000 kb. All selected SNPs were required to have a minor allele frequency (MAF)>1%. We also searched through PhenoScanner GWAS database (http://phenoscanner.medschl.cam.ac.uk, Version 2) and removed previously reported SNPs (if existed) associated with the outcome and its known confounders under a genome-wide significance threshold of P < 5 ×10^-8^[13]. |
|  | d) | For each exposure, outcome, and other relevant variables, describe methods of assessment and diagnostic criteria for diseases | The diagnosis of EoE was both clinically and pathologically confirmed. Detailed information regarding definition of cases, genotype platforms and statistical analysis protocols are available at the FinnGen website [https:/www.finngen.fi/en/]. |
|  | e) | Provide details of ethics committee approval and participant informed consent, if relevant | The study was approved by the corresponding ethics committee and informed consents were collected from all participants. |
| 5 | **Assumptions** | Explicitly state the three core IV assumptions for the main analysis (relevance, independence and exclusion restriction) as well assumptions for any additional or sensitivity analysis | Of note, three assumptions need to be fulfilled in a compelling MR study. First, IVs should be strongly associated with the exposure, which is guaranteed here by the threshold of P<5 ×10-8 in the creation of IVs. Second, IVs should influence outcomes through risk factors and not through any direct causal pathway. Third, IVs are not associated with any known or unknown confounders[21] |
| 6 | **Statistical methods: main analysis** | Describe statistical methods and statistics used |  |
|  | a) | Describe how quantitative variables were handled in the analyses (i.e., scale, units, model) | We used the random-effect inverse variance weighted (IVW) method to calculate the primary result[15]. We used MR-egger and weighted median methods to test the robustness of our primary result[15, 16]. Estimates of individual SNP-exposure correlation versus SNP-outcome correlation were visualized by scattered plots. Heterogeneity was assessed using the Cochran’s Q test and was intuitively shown by the leave-one-out analysis[14]. Meta-analyses of MR results were conducted by using a random-effect model in the Revman software (Version 5.3.3). Heterogeneity across MR results were evaluated using the Cochrane chi-square and quantified with the I2 value. I2 values of 25, 50 and 75% represent low, moderate and high heterogeneity, respectively[20]. The reverse MR applied similar analytic strategy, the exposure GWAS of IBD, UC and CD were ieu-a-31, ieu-a-32 and ieu-a-30, respectively. |
|  | b) | Describe how genetic variants were handled in the analyses and, if applicable, how their weights were selected | NA |
|  | c) | Describe the MR estimator (e.g. two-stage least squares, Wald ratio) and related statistics. Detail the included covariates and, in case of two-sample MR, whether the same covariate set was used for adjustment in the two samples | We used the random-effect inverse variance weighted (IVW) method to calculate the primary result [15]. We used MR-egger and weighted median methods to test the robustness of our primary result [15, 16]. Estimates of individual SNP-exposure correlation versus SNP-outcome correlation were visualized by scattered plots. |
|  | d) | Explain how missing data were addressed | NA |
|  | e) | If applicable, indicate how multiple testing was addressed | We used MR-egger and weighted median methods to test the robustness of our primary result [15, 16]. |
| 7 | **Assessment of assumptions** | Describe any methods or prior knowledge used to assess the assumptions or justify their validity | The significant threshold was set as P<5 ×10^-8^ to filter single nucleotide polymorphisms (SNPs) strongly correlated with the exposure. We also searched through PhenoScanner GWAS database and removed previously reported SNPs (if existed) associated with the outcome and its known confounders under a genome-wide significance threshold of P < 5 ×10^-8^[13]. |
| 8 | **Sensitivity analyses and additional analyses** | Describe any sensitivity analyses or additional analyses performed (e.g. comparison of effect estimates from different approaches, independent replication, bias analytic techniques, validation of instruments, simulations) | When significant heterogeneity was detected, three sensitivity analyses were further conducted. The MR pleiotropy residual sum and outlier (MR-PRESSO) method was utilized to identify and to exclude outliers of instrumental variables with significant pleiotropic effect [17] (sensitivity analysis A). The MR-Radial analysis was further performed as sensitivity analysis B to identify and to exclude SNPs which were major source of heterogeneity [18, 19]. The sensitivity analysis C excluded SNPs in both sensitivity analysis A and B. |
| 9 | **Software and pre-registration** |  |  |
|  | a) | Name statistical software and package(s), including version and settings used | MR analyses were performed by using the TwoSampleMR R package and a series of ancillary packages in the R software (Version 4.2.1)[14]. |
|  | b) | State whether the study protocol and details were pre-registered (as well as when and where) | NA |
|  | **RESULTS** |  |  |
| 10 | **Descriptive data** |  |  |
|  | a) | Report the numbers of individuals at each stage of included studies and reasons for exclusion. Consider use of a flow diagram | The study flowchart is presented in Figure 1. |
|  | b) | Report summary statistics for phenotypic exposure(s), outcome(s), and other relevant variables (e.g. means, SDs, proportions) | Altogether, 15 independent SNPs correlated with EoE were filtered, none of which has been previously identified as the genetic loci associated with IBD, CD or UC. Ten of the screened SNPs were selected from the FinnGen dataset and five SNPs with a MAF<1% were excluded (Table S1). |
|  | c) | If the data sources include meta-analyses of previous studies, provide the assessments of heterogeneity across these studies | In the meta-analysis of estimates from IVW, the pooled OR was 1.07 (95% CI, 1.02-1.13; I2=0, Figure 3A). No significant result was obtained from meta-analyses of MR-Egger (OR, 0.95; 95% CI, 0.79-1.14; I2=0, Figure 3B) and weighted median (OR, 1.03; 95% CI, 0.99-1.07; I2=0) methods (Figure 3C). |
|  | d) | For two-sample MR:  i.  Provide justification of the similarity of the genetic variant-exposure associations between the exposure and outcome samples  ii.  Provide information on the number of individuals who overlap between the exposure and outcome studies | i: The EoE dataset originated from a meta-analysis of GWAS. The open-source meta-analysis included 1,930 patients with EoE and 13,634 controls of European ancestry. The GWAS summary data of IBD and its subtypes were obtained from the latest FinnGen datasets which contain 9,083 IBD, 2,033 CD and 5,931 UC patients of European ancestry, respectively.  ii: There is no participant overlap between the exposure and outcome datasets. |
| 11 | **Main results** |  |  |
|  | a) | Report the associations between genetic variant and exposure, and between genetic variant and outcome, preferably on an interpretable scale | Estimates of individual SNP-EoE correlation versus SNP-IBD correlation were visualized in Figure 2A. |
|  | b) | Report MR estimates of the relationship between exposure and outcome, and the measures of uncertainty from the MR analysis, on an interpretable scale, such as odds ratio or relative risk per SD difference | eg: For the MR using FinnGen dataset, the result indicated a causal relationship between EOE and IBD (odds ratio (OR), 1.07; 95% confidence interval [CI], 1.01-1.13). |
|  | c) | If relevant, consider translating estimates of relative risk into absolute risk for a meaningful time period | NA |
|  | d) | Consider plots to visualize results (e.g. forest plot, scatterplot of associations between genetic variants and outcome versus between genetic variants and exposure) | eg: Estimates of individual SNP-EoE correlation versus SNP-IBD correlation were visualized in Figure 2A; Significant heterogeneity was detected by the Cochrane’s Q test (Q=17.9; P=0.022) and is visualized by the plot of leave-one-out analysis (Figure S2A). |
| 12 | **Assessment of assumptions** |  |  |
|  | a) | Report the assessment of the validity of the assumptions | Of note, three assumptions need to be fulfilled in a compelling MR study. First, IVs should be strongly associated with the exposure, which is guaranteed here by the threshold of P<5 ×10-8 in the creation of IVs. Second, IVs should influence outcomes through risk factors and not through any direct causal pathway. Third, IVs are not associated with any known or unknown confounders[21]. To verify the last two assumptions, we have searched through the PhenoScanner GWAS database to check whether there is a reported relationship between selected SNPs and the outcome as well as its known confounders. The IVs in the forward MR are mainly associated with atopy, which has not been proven to correlate with IBD and its confounding factors like connective tissue disease, infection, antibiotics use, smoking and diet. Also, IVs utilized in the reverse MR has not been found to be involved in EoE and its known risk factors like food allergy and aeroallergens. |
|  | b) | Report any additional statistics (e.g., assessments of heterogeneity across genetic variants, such as *I^2^*, Q statistic or E-value) | eg: Significant heterogeneity was detected by the Cochrane’s Q test (Q=17.9; P=0.022) and is visualized by the plot of leave-one-out analysis (Figure S2A). One pleiotropic outlier was detected by MR-PRESSO (rs56062135, P=0.019). The MR-Radial analysis further identified one outlying SNP. The sensitivity analysis results after removing outliers were demonstrated in Table S3. |
| 13 | **Sensitivity analyses and additional analyses** |  |  |
|  | a) | Report any sensitivity analyses to assess the robustness of the main results to violations of the assumptions | Significant heterogeneity was detected by the Cochrane’s Q test (Q=17.9; P=0.022) and is visualized by the plot of leave-one-out analysis (Figure S2A). One pleiotropic outlier was detected by MR-PRESSO (rs56062135, P=0.019). The MR-Radial analysis further identified one outlying SNP. |
|  | b) | Report results from other sensitivity analyses or additional analyses | eg: The sensitivity analysis results after removing outliers were demonstrated in Table S3. |
|  | c) | Report any assessment of direction of causal relationship (e.g., bidirectional MR) | eg: Consistently, results of IVW (OR, 1.08; 95% CI, 1.02-1.14) and weighted median (OR, 1.07; 95% CI, 1.02-1.13) methods indicated a positive causality of EoE on UC (Figure 2C, Figure S1C).  No causal link was found by IVW (OR, 0.98; 95% CI, 0.89-1.08), MR-Egger (OR, 1.02; 95% CI, 0.73-1.43) and weighted median (OR, 1.02; 95% CI, 0.90-1.16) methods (Figure 4B, Figure S1G). |
|  | d) | When relevant, report and compare with estimates from non-MR analyses | NA |
|  | e) | Consider additional plots to visualize results (e.g., leave-one-out analyses) | Significant heterogeneity was detected by the Cochrane’s Q test (Q=17.9; P=0.022) and is visualized by the plot of leave-one-out analysis (Figure S2A). |
|  | **DISCUSSION** |  |  |
| 14 | **Key results** | Summarize key results with reference to study objectives | Using publicly available GWAS summary statistics, results of our bidirectional two-sample MR analysis suggested a possible causal association of EoE on IBD, especially on UC (the risk effect is minimal in CD). Genetic liability to IBD or any subtype was not found to correlate with EoE in our analyses. |
| 15 | **Limitations** | Discuss limitations of the study, taking into account the validity of the IV assumptions, other sources of potential bias, and imprecision. Discuss both direction and magnitude of any potential bias and any efforts to address them | This study inevitably has several limitations. First, as an ethnicity-limited study, whether the findings could be generalized to other ethnic populations remains unclear. With the update of EoE GWAS from other populations, the validation analysis should be conducted to test the robustness of our findings in different populations. Second, the GWAS data of EoE were generated based on children and adolescent patients. Though no evidence of difference in genetic variants between pediatric and adult EoE patients has been generated, selection bias could not be fully excluded. Third, an absence of detailed information regarding severity stratification, status and duration of IBD and its subtypes in both FinnGen and IIBDGC GWASes limited the evaluation of selection bias and the performance of further subgroup analyses. Variations in clinical phenotypes, disease severity and clinical outcome are observed in IBD, and these have been linked to underlying genetic basis [36, 37]. Additionally, we preliminarily assessed the genetic overlap between EoE and IBD (data from the ieu-a-31 GWAS dataset) via the linkage disequilibrium score regression [38], and the result showed no evidence of a genetic correlation between the two diseases (genetic correlation=-0.325, P=0.393). Generally, a consistency between results of linkage disequilibrium score regression and MR could made the MR estimates more convincing [39]. Therefore, results generated by this study should still be interpreted with caution. Further studies, especially EoE GWASes with larger sample size are warranted to resolve the above issues. |
| 16 | **Interpretation** |  |  |
|  | a) | Meaning: Give a cautious overall interpretation of results in the context of their limitations and in comparison with other studies | Therefore, results generated by this study should still be interpreted with caution. Further studies, especially EoE GWASes with larger sample size are warranted to resolve the above issues. |
|  | b) | Mechanism: Discuss underlying biological mechanisms that could drive a potential causal relationship between the investigated exposure and the outcome, and whether the gene-environment equivalence assumption is reasonable. Use causal language carefully, clarifying that IV estimates may provide causal effects only under certain assumptions | Concurrent diagnosis of EoE and IBD has attracted attention clinically, and an exclusion of IBD is recommended when a diagnosis of EoE is made [23].The underlying mechanism remains ambiguous, and several explanations have been proposed. Although involved regions locate at the opposite end of the GI tract, EoE and UC both invoke Th-2 mediated pathways[24, 25] with shared pro-inflammatory cytokines (mainly interleukin-5 and interleukin-13) and shared activation of downstream Janus kinase and signal transducer and activator of transcription (JAK-STAT) pathways (mainly STAT 3 and STAT 6) [26-28].That is to say, an early overactivation of Th-2 immune response in EoE patients may trigger a subsequent development of UC. On the contrary, CD is mainly mediated by Th-1 cellular immune response and thus shows a weaker link with EoE. Besides the adaptive immune response, pathogenesis of both diseases involves upregulation of toll-like receptors, a critical class of proteins in the innate immune system [29, 30]. The activation of innate immune response against GI bacteria triggers inflammation in both esophageal and intestinal mucosa. An additional explanation is the impairment of epithelial barrier function and exposure to shared pathogenic environmental factors in both diseases. Elevated interleukin-13 in both diseases down-regulate proteins associated with barrier function (desmoglein-1 and filaggrin, etc) and altered epithelial permeability in the GI tract [31-33], permitting interactions between risk environmental factors (food antigens, microbial dysbiosis, antibiotic use, etc) and esophageal/intestinal immune system [28, 34, 35]. Aberrant immune responses against these antigens provoke mucosal inflammation and cause diseases. |
|  | c) | Clinical relevance: Discuss whether the results have clinical or public policy relevance, and to what extent they inform effect sizes of possible interventions | Herein, we innovatively conduct a bidirectional two-sample MR study to evaluate the causal relationship between EoE and IBD, providing new insights into the understanding the high comorbidity between these two immune-mediated gastrointestinal diseases. Clinically, the present findings call for an increased awareness of concurrent or subsequent IBD, especially UC, in the management of patients with EoE. Besides regular gastroscopy, colonoscopy might be taken into consideration during the follow-up of EoE patients. |
| 17 | **Generalizability** | Discuss the generalizability of the study results (a) to other populations, (b) across other exposure periods/timings, and (c) across other levels of exposure | First, as an ethnicity-limited study, whether the findings could be generalized to other ethnic populations remains unclear. With the update of EoE GWAS from other populations, the validation analysis should be conducted to test the robustness of our findings in different populations. Second, the GWAS data of EoE were generated based on children and adolescent patients. Though no evidence of difference in genetic variants between pediatric and adult EoE patients has been generated, selection bias could not be fully excluded. Third, an absence of detailed information regarding severity stratification, status and duration of IBD and its subtypes in both FinnGen and IIBDGC GWASes limited the evaluation of selection bias and the performance of further subgroup analyses. Variations in clinical phenotypes, disease severity and clinical outcome are observed in IBD, and these have been linked to underlying genetic basis [36, 37]. |
|  | **OTHER INFORMATION** |  |  |
| 18 | **Funding** | Describe sources of funding and the role of funders in the present study and, if applicable, sources of funding for the databases and original study or studies on which the present study is based | The study is funded by the CAMS Innovation Fund for Medical Sciences (grant number, CIFMS 2021-I2M-1-003). The funder is not involved in study design, data collection, data analysis and interpretation of results. |
| 19 | **Data and data sharing** | Provide the data used to perform all analyses or report where and how the data can be accessed, and reference these sources in the article. Provide the statistical code needed to reproduce the results in the article, or report whether the code is publicly accessible and if so, where | The original contributions are included in the article/Supplementary Material. Further inquiries can be directed to the corresponding authors. |
| 20 | **Conflicts of Interest** | All authors should declare all potential conflicts of interest | The authors declare that they have no relevant conflicts of interest. |

This checklist is copyrighted by the Equator Network under the Creative Commons Attribution 3.0 Unported (CC BY 3.0) license.

1. Skrivankova VW, Richmond RC, Woolf BAR, Yarmolinsky J, Davies NM, Swanson SA, et al. Strengthening the Reporting of Observational Studies in Epidemiology using Mendelian Randomization (STROBE-MR) Statement. JAMA. 2021;under review.

2. Skrivankova VW, Richmond RC, Woolf BAR, Davies NM, Swanson SA, VanderWeele TJ, et al. Strengthening the Reporting of Observational Studies in Epidemiology using Mendelian Randomisation (STROBE-MR): Explanation and Elaboration. BMJ. 2021;375:n2233.
